# Part I: determination of a structure/property transformation mechanism responsible for changes in the point of zero change of anatase titania with decreasing particle size

**DOI:** 10.1039/d4ra01139b

**Published:** 2024-10-15

**Authors:** Miriam Leffler, Anne Mirich, Jared Fee, Seth March, Steven L. Suib

**Affiliations:** a Department of Chemistry, University of Connecticut USA Miriamleffler1066@gmail.com anne.mirich@uconn.edu jare.fee@uconn.edu seth.march@uconn.edu steven.suib@uconn.edu

## Abstract

Below a diameter of approximately 28 nm, the surface crystal structure of anatase titania is known to change. These changes include surface bond lengths and crystal lattice parameter expansion/contractions. Concurrent with these structure changes, the materials point of zero charge (PZC) has been observed to shift toward lower pH values. Therefore, the objective of this work was to determine if a correlation exists between these known structural changes and the shift in the materials PZC values with decreasing particle size. To achieve this a method was developed to identify and minimize the effect of all known variables, save particle size, affecting the materials pH_PZC_. This led to the discovery of two regions for point of zero charge. Above the average spherical primary particle diameter ≅ 29 nm for anatase titania, denoted as Region I, PZC values remain constant. In Region I the materials surface crystal structure and properties were also found to remain constant. Below the average spherical primary particle diameter ≅29 nm is the second zone, defined as Region II, where pH_PZC_ values decrease almost linearly. An examination of possible surface structure factors and properties responsible for the shift in these PZC values (Region II) identified three underlying causes. These being changes in the materials band gap (*i.e.* surface bond lengths), lattice parameters and bond ionic content.

## Abstract 2

Below a diameter of approximately 28 nm, the surface crystal structure of anatase titania is known to change.

## Graphical abstract

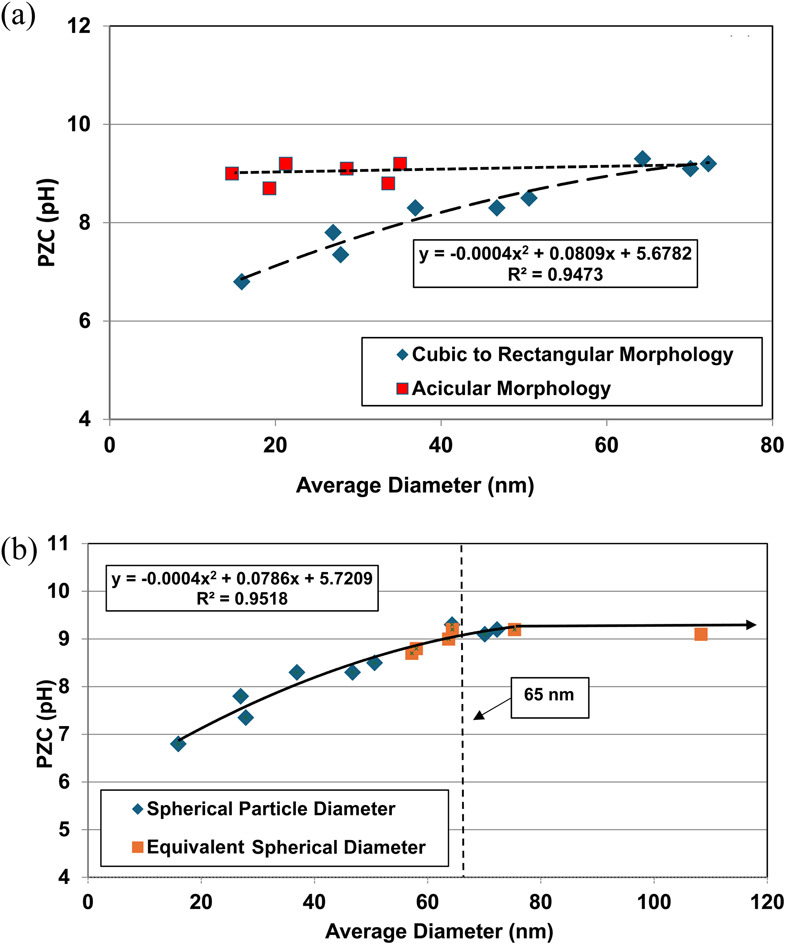

## 1 Introduction

Point of zero charge (PZC) is an important adsorption property, critical to multiple areas in chemistry where the surface charge is neutralized by counter ions resulting in its surface potential going to zero. In catalysis PZC has been found to control the kinetics of electrochemical reduction of diazonium salts.^1^ Changes in surface roughness have been identified as shifting the material's isoelectric point/PZC affecting its utility as a catalyst support.^2^ PZC is also critical to the loading and retention (*i.e.* adhesion) of a catalyst onto a support.^3^

Changes in PZC values are also important in their effect on dielectric and capacitive properties, which affect electrode performance.^4,5^ These changes can occur through the introduction of different protons and specifically adsorbing ions^6^ at the solid/liquid interface.^7–10^ The effect on these properties is important in electrical energy storage devices such as batteries and capacitors, which are critical in electronic systems.^11,12^

PZC is in fact critical across many other fields of chemistry beyond the two detailed above. These include flotation in mineral engineering (*i.e.*, extracting valuable minerals from ores),^13^ waste remediation (contaminate removal),^14^ soil science,^15^ electrochemistry,^16^ pharmacology (drug production),^17^ colloidal chemistry and surface science.^18^ Therefore, if only small changes to the surface structure have a significant effect on this property, a fundamental understanding of the underlying factor(s) controlling a material's PZC value is critical. It will serve to advance work in these and other fields of chemistry where this value is important to their work.

Anatase titania was chosen for this work due to its importance in multiple fields. These include water purification systems,^19^ photocatalysis,^20^ electrochemical and biosensors,^21^ a finishing agent on cloth allowing it to self-clean,^22^ and photovoltaic systems.^23^ Changes in these materials' point of zero charge (PZC) in the synthesized material have been shown to affect properties such as photocatalysis,^24^ water treatment,^25^ and catalytic reaction rates.^26^ Therefore, the underlying cause(s) responsible for the shift in this property will be important across multiple fields where this material is used.

The multiple factors affecting PZC/surface charge have been studied and identified in the literature. These include:

(1) Morphology^2^

(2) Roughness^2^

(3) Particle size^27,28^

(4) Surface crystal structure^7–9^

(5) Composition^10^

(6) Temperature^29^

(7) Surface contamination/surfactants^30^

(8) Representative sample size of particle population^31^

(9) Solubility^32^

(10) Pretreatment, which can introduce solvents, acids, bases *etc.* into the pore system, changing the overall composition of the surface, resulting in a shift in the PZC value^10,33^

(11) Grinding^34^

(12) Stable surface phase (*i.e.*, dominant species) in an aqueous environment at its PZC value according to its Eh-pH diagram,^35^ and

(13) Electrolyte concentration^36^

Yet even with knowledge of these variables, PZC values for the same phase often extend over 3 to 4 pH units.^9,37,38^ Therefore, an understanding of how each of these factors impinges on PZC values is critical.

Theoretical work by Barisik *et al.*^28^ determined that as spherical silica nanoparticle's size decreased below *d* ≅ 20 nm, surface charge increased significantly. Above this diameter, surface charge remained essentially constant. Their work allowed the examination of changes in a particles surface charge without the physical constraints cited above. Therefore, decreasing particle size would allow an examination of surface structure/property changes to be examined once the known factors affecting pH_PZC_ are minimized.

Work carried out by Suttiponparnit *et al.*^39^ demonstrates the difficulty of determining an accurate primary particle size. They used the specific surface area of each sample to determine the average primary particle size for each PZC value of anatase titania. Their results though were opposite those found by Barisik *et al.*^28^ They observed that as the surface area increased (*i.e.* decreasing particle size) PZC values were found to increase. Yet even with this discrepancy between their results and Barisik *et al.*'s,^28^ the importance of their work is that they demonstrated experimentally that there is a systematic shift in anatase titania's PZC with changing surface area (*i.e.*, particle size) and was the impetus for this research.

Defining a normalized average primary particle diameter even for the same powder population measured presents a significant problem. Lin *et al.*'s^40^ work illustrates this. Their work used three different methods to measure an average spherical particle diameter (*D*_SP_) for the same sample of anatase titania. These included hydrodynamic diameter (H. D.), BET (surface area using nitrogen adsorption), and transmission electron microscopy (TEM). The H. D. method resulted in a *D*_SP_ = 314 ± 8 nm, while TEM gave a *D*_SP_ = 11.0 ± 3.4 nm. Using the equation for a spherical diameter [ref. 41, Appendix A], the specific surface area (*S*_A_) from the BET (324 m^2^ g^−1^) and the density of anatase titania (*ρ* = 3.89 g cm^−3^)^42^ gave an average primary particle *D*_SP_ = 4.75 nm for a particle with a spherical, cubic, and/or rectangular morphology.

Each of the preceding examples demonstrates the difficulty of obtaining a normalized average primary particle size for a powder population. Once a method for normalizing each powder population's average primary particle diameter is achieved, it should then be possible to determine the correct shift in a material's PZC values with size. Furthermore, this would allow an examination of the underlying changes to a material's surface structure and properties for these changes.

Leffler^43^ addressed this problem by devising a method to standardize/normalize the average primary particle size (*i.e.*, diameter) between two separate populations of the same phase. The material chosen to test this method was goethite (α-FeO(OH)). Synthesized goethite used in experimental work possesses two morphologies, these being: (1) cubic to rectangular and (2) acicular (needle like).^44–46^

**Fig. 1:**
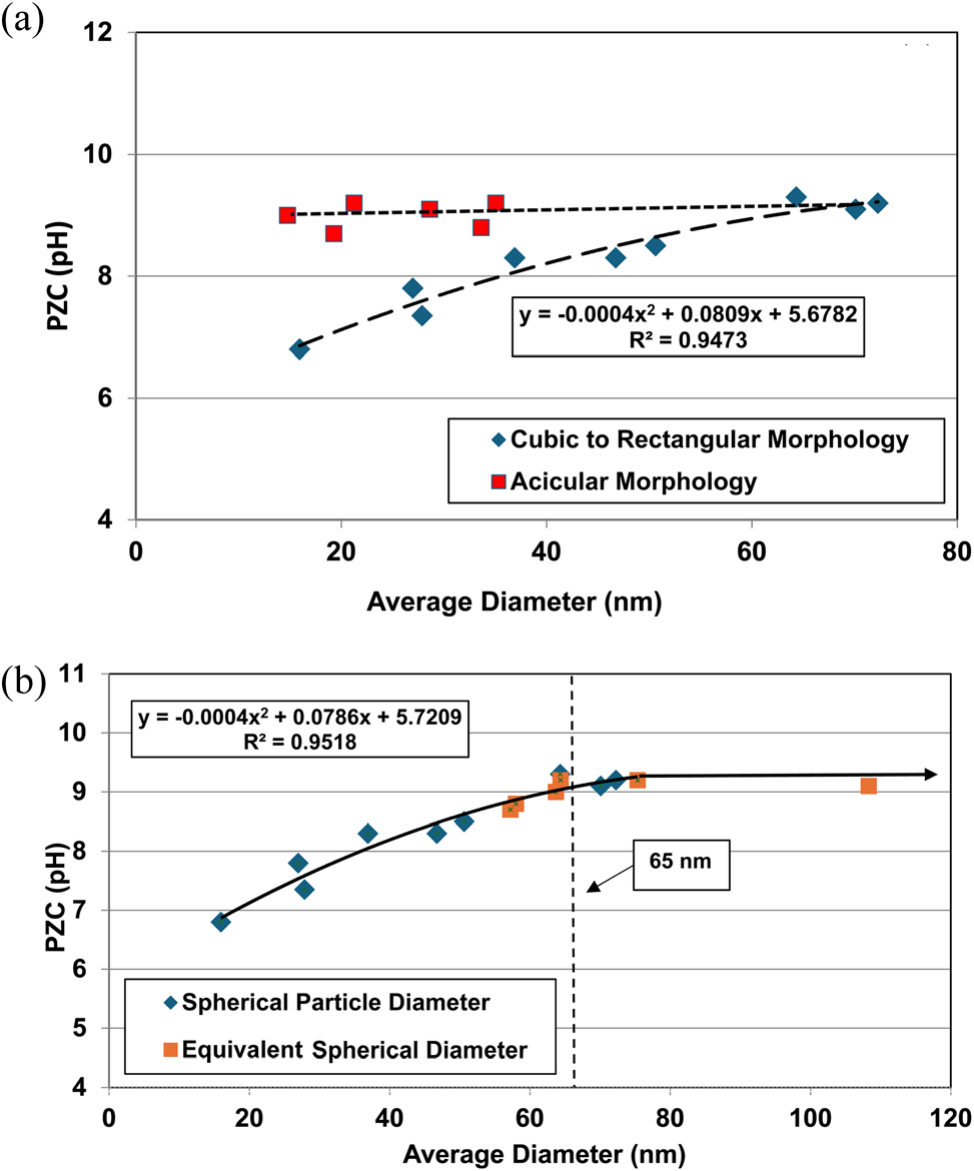
(a) Correlation between the calculated average primary particle spherical diameters using specific surface area plotted against their PZC values for Groups 1 & 2 of synthetic goethite (α-FeO(OH)).^47–59^ (b) Correlation between the spherical to rectangular particle diameters with the normalized acicular particle diameters against their PZC values for Groups 1 & 2 of goethite (α-FeO(OH)).^47–59^

The effect of these two different morphologies, where their size (*i.e. D*_SP_) has not been normalized, is presented in Fig. 1a. Using the equation for a spherical diameter [ref. 41, Appendix A], two sets of particle sizes for these goethite groups were calculated, using their specific surface area. Group 1 possesses a morphology that is roughly cubic to rectangular^47–55^ which decreases almost linearly below a *d* ≅ 65 nm. Group 2 possesses an acicular (*i.e.*, needle like) morphology^56–59^ which demonstrates no change in the material's pH_PZC_ values with decreasing particle size. In Fig. 1a there is no apparent correlation between group 1 & 2.

The next step was to calculate the equivalent spherical radius (*R*_ES_) for Group 2 using each population's acicular volume.^60^ Once the equivalent particle diameters (*D*_ES_ = 2 × *R*_ES_) were normalized and plotted along with the values from group 1, a single curve emerged and is presented in Fig. 1b. The single curve decreases almost linearly for both particle populations below *d* ≅ 65 nm. This curve now allows correlation between any possible underlying structure and/or property parameter(s) responsible for these changes in PZC values without the complicating factor of the material's average primary particle morphology. Appendix A provides the full method used to obtain the results in Fig. 1a and b.

The method used to obtain the results in Fig. 1b was also used to determine the average primary particle size (*D*_SP_) for other metal oxides/hydroxides from literature values. These values were then plotted against their PZC values. The results obtained are available in Appendix A. They include barium titanate (BaTiO_3_),^61–72^ monoclinic zirconia (ZrO_2_),^73–85^ hematite (α-Fe_2_O_3_),^86–95^ and 3% doped tetragonal zirconia (3% YTZ).^96–109^ They follow the same form as the curve in Fig. 1b. The final plot [Fig. 12] demonstrates the effect of the largest cation^110^ in each structure on the particle size at which PZC values begin shifting to lower pH_PZC_ values.

Until this work there has been no systematic method for identifying and minimizing the effect of all the known possible variables responsible for changes in each metal-oxide/hydroxide point of zero charge values.^2,7–10,27–36^ Typically, studies into the effect of different variables of point of zero charge often consider only a few of the factors responsible for changes in this property. Therefore, the objectives of this project were to (1) develop a method minimizing the effect of all the known factors affecting PZC values for all metal-oxides/hydroxides, (2) develop a protocol for obtaining the most accurate and precise normalized average primary particle diameters of different morphologies with the same phase and (3) determine any underlying physical parameter(s) and/or properties which correlate directly with any changes in the materials pH_PZC_.

## 2 Experimental section

### 2.1 Choice of metal oxide systems

Anatase titania [US Research Nanomaterials, SkySpring Nanomaterials] was chosen for the following reasons: (1) the phase is stable at ambient temperature and pressure,^111^ (2) the material is available commercially from the micron region down to a diameter of 5 nm,^112,113^ (3) it is stable even when exposed to corrosive environments,^114^ (4) is highly insoluble over a wide range of pH values in an aqueous environment, dilute acids and inorganic bases at ambient temperature and pressure,^3,42,115^ (5) the bulk and surface structures are identical,^9^ and (6) the dominant species is TiO_2_ from a pH = 0 to 14 in an aqueous environment at ambient temperature and pressure^35^ indicating there is no thermodynamic driving force to transform the surface structure, allowing it to remain stable.

### 2.2 Determination of the average primary particle size, morphology, roughness and elemental composition

The samples obtained exhibited a spherical to cubic morphology, with smooth surfaces in the TEM micrographs available from the vendor for each sample tested.^112,113^ Detailed particle morphology, size and surface smoothness were then reconfirmed using an FEI, Talos F200× microscope utilizing a 200 kV voltage transmission electron microscope (TEM). Elemental mapping was done with an energy dispersive X-ray spectroscopy (EDS) detector. The EDS is sensitive down to 0.1% by weight of elements present in the sample. These include even light elements such as carbon, nitrogen and oxygen^116^ which can be component parts of solvents. The TEM grids were prepared by combining a 2 mg sample with 20 mL of solvent (ethanol: Fischer Scientific) and sonicating for 5 minutes. The solution was then dispersed on a copper grid dropwise *via* a pipette. Multiple micrographs were taken of each powder population. Representative TEM micrographs of each sample are presented in Appendix B.

### 2.3 Minimum sample size

To ensure a representative sample size in each literature and experimental data set, eqn (1) through (3) were used to calculate an absolute theoretical minimum amount (*M*_*i*_).^31,42^1
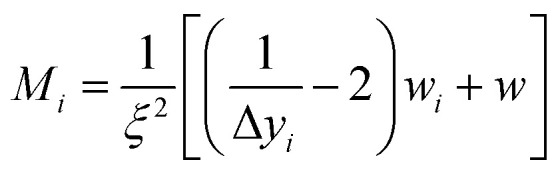

2 *w*_*i*_ = (*k*_*υ*_*d*^3^*ρ*)

3
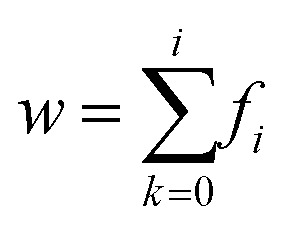
where *ξ* = the maximum allowable error (%), Δ*y*_*i*_ = mass of fraction (g), *k*_*ν*_ = shape factor (6 π^−1^), *ρ* = density (g cm^−3^), *d* = particle diameter (nm).

Since *M*_*i*_ typically corresponds closely to the top size of the distribution, and only this value was calculated. For this material a *d* = 1 µm was chosen which was significantly larger than each of the average primary particle sizes in the powder populations examined. In addition, as the diameter of *M*_*i*_ decreases, the sample needed for a representative sample also decreases. Therefore, the calculated *M*_*i*_ = 1 µm would be significantly larger than required for the average primary particle diameters used. The variables input into eqn (3) through (5) were for a typical Rosin-Rammler size distribution, with a slope = 1, with *ξ* = ± 5% (0.05) maximum allowable error, and the top fraction size is 10% (0.10) for a 1.0 g powder population, where Δ*y*_*i*_ = 0.10 g. The density used for anatase titania was *ρ* = 3.84 g cm^−3^.^42^ The minimum top size value calculated for anatase titania [eqn (3)–(5)] is *M*_*i*_ = 2.96 × 10^−11^ g.

### 2.4 Confirmation of crystal structure of anatase titania using X-ray diffraction

Powder X-ray diffraction (PXRD) patterns were taken to confirm both the structure and composition of all seven powder samples. PXRD micrographs of each sample [Appendix C] were made using a Rigaku Ultima IV X-ray diffractometer set at 40 kV, 44 mA, and 1.76 kW. The system is sensitive down to 0.2% by weight^117^ of all structures present in the sample. The PXRD patterns were taken between 5° to 75° 2-theta. The CuKα1 X-rays were produced using a copper tube with a wavelength of *λ* = 1.54056. Rigaku PDXL 1.8.0.3 software was used to analyze the structures.

### 2.5 Temperature range

The ambient temperature range within which each measurement was taken ran from 293 K to 298 K. This was to minimize the temperature effect on the measured PZC value.^29^ The solution temperature was monitored and recorded during the titration using a temperature probe on a Hanna Instruments (HI) 2214 pH/ORP meter. This insured that during the titration the electrolyte solution remained within the designated temperature range.

### 2.6 Prevention of contamination and no pretreatment

To avoid contamination each sample remained in its container until used in its titration run. There was no pretreatment of the sample, as this is known to affect measured PZC values.^27^ This procedure reduced the possibility of surface contamination to a minimum before testing.

### 2.7 Mass titration method

The mass titration method was used to determine PZC values of anatase titania samples which is comparable to the common intercept point (CIP). An added advantage of this method is that avoids the possible problems posed using different electrolyte concentrations employed in CIP.^118^ The procedure involved performing three runs, one with a blank (0.0 g sample) electrolyte solution and two more runs with increasing powder sample sizes. The full procedure is presented in Appendix D.

### 2.8 Band gap measurements

Band gap measurements were made thru Eurofins Materials Science at the EAG Laboratories in Eindhoven, the Netherlands. Two sets of samples were measured. The samples presented in this paper were pressed in an XRD sample holder by hand (*i.e.*, at ambient temperature and pressure) and a second set of powder samples, pressed at 10 tons. A PerkinElmer, Lambda 1050 UV-VIS-NIR spectrometer equipped with a 150 mm integrating sphere was used to perform the measurements. Reflection UV-VIS-NIR analysis was performed between 200 and 1000 nm. Eqn (4) was used to calculate band gap energy.

4 Band gap energy (*E*) = *h* × *C*/*λ*

where *h* = Planks constant = 6.626 × 10^−34^ J s, *C* = speed of light = 3.0 × 10^8^ m s^−1^, *λ* = cut off wavelength, with 1 eV = 1.6 × 10^−19^ joules (conversion factor).

Fig. 28, in Appendix E, presents the plot of band gap values for the sample pressed at 10 tons. An explanation of the effect pressure has on the materials surface bond lengths is included.^119^ Therefore, the set of measurements taken at ambient temperature and pressure were used. This allowed the comparison of the band gap results with all the other experimental work carried out under the same conditions at ambient temperature and pressure.

## 3 Results

### 3.1 Powder population average primary particle size and elemental composition

An examination of the particles for each powder population using transmission electron microscopy (TEM) micrographs revealed four basic shapes. These were a circle (sphere), ellipse (ovoid), square (cube) and rectangle (rectangular cube) [Appendix B]. The method developed by Leffler^43^ was then augmented to determine the average equivalent spherical diameter (*D*_ES_).

The first step was to identify and physically outline the particles so their surface was clearly identified, and their entire circumference could be easily measured. Next, circular (spherical) particle diameters (*D*_S_) were then measured directly from the TEM micrographs. The other three non-circular particles were determined by calculating their equivalent circular radius (*R*_ES_). This was accomplished by first obtaining the surface area equations for a circle (*A* = *r*^2^ × π), ellipse (*A* = *q* × *p* × π), square (*A* = *a*^2^), and rectangle (*A* = *w* × *h*).^60^ Then the surface area equations for the three non-circular shapes were set equal to the surface area of the circle. Rearranging these equations resulted in the equations for the *R*_ES_ of each non-circular shape. Then inserting *R*_ES_ into *D*_ES_ = 2 × *R*_ES_ gave eqn (5) through (7).5
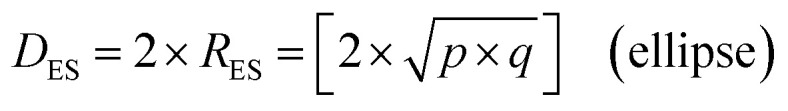
6
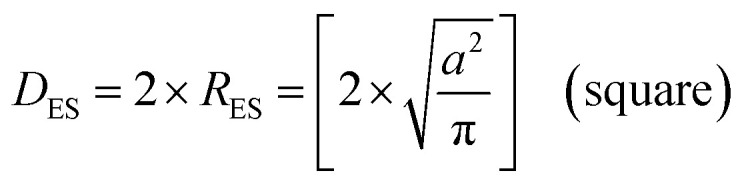
7
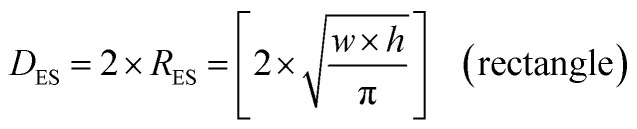
where *r* = radius (nm), *p* = semi minor axis of an ellipse (nm), *q* = semi-major axis of an ellipse (nm), *a* = length of square side of square (nm), *w* = width of a rectangle (nm), *h* = height of a rectangle (nm).

The next step was to find the sample size required to obtain an accurate average *D*_ES_ for each powder population. This was done by calculating the average diameter for a series of increasing sample sizes. An example of this process is presented in Table 1 for powder population 7. The first average *D*_ES_ was determined for a sample size of 5 measured particle diameters. This gave an average *D*_ES_ = 121.77 nm. Then the sample size was increased to 10 particles and the process repeated, each time incrementing the sample size by 10 particles. The reason for using powder population 7, is that this one demonstrated the widest variation of measured particle sizes, stretching from a *D*_ES_ ≅ 56.45 nm to a *D*_ES_ ≅ 256.41 nm.

**Table 1:** Average primary particle diameter with increasing sample size for powder population 7

Sample size	Average particle diameter (nm)	Average particle variation (±nm)
5	121.77	22.30
10	151.30	33.12
20	151.98	31.71
30	147.27	35.68
40	143.33	35.32
50	140.95	32.78
60	139.98	30.69
70	141.77	31.72
80	142.614	31.66
90	142.45	31.75
100	143.11	33.59
110	144.35	35.02
120	143.45	35.84
127	142.614	36.32

It was found that above a sample size of 50, the average diameters obtained for sample sizes from 50–127 were found to oscillate around an average *D*_ES_ = 142.233 nm with an average variation of ±1.021 nm [Table 1]. This indicates that the sample size required to obtain the most accurate value is at/or above 50 spherical and equivalent spherical particle diameters. Sample sizes for all the powder populations examined in this work far exceeded this, typically about 100 particles were measured. Results for all the powder populations are presented in Appendix B, in Table 3.

In addition to the average primary particle diameters determined from TEM measurements, are two additional sets of particle sizes for each powder population in Table 3 [Appendix B]. This data was available on the websites for both U. S. Nanomaterial Research^112^ and Spring Sky Nanomaterials.^113^ The average primary particle diameters were determined from their BET (nitrogen adsorption) measured specific surface area (m^2^ g^−1^) using eqn (13), and *ρ* = 3.84 g cm^−3^.^45^ The second method was an Aerodynamic Particle Sizer (APS) which relies on time of flight coupled with Dynamic Light Scattering (DLS) to measure particle size. A comparison of the results from the three methods for the powder populations, using identical synthesis process was made. The differences between the average primary particle size for the method developed in this work and those provided by the companies^112,113^ ranged from 5.785% to 463.301%, with an average of 66.355% for all the values in the two sets.

A plot of both sets of the experimental particle data against their PZC values (unpublished) from both vendors [Table 3] was made for their Region II values. The best fit function for the curves was a second order polynomial. The correlation factors (*R*^2^) for these two curves gave values of *R*^2^ = 0.4567 (BET) and *R*^2^ = 0.5337 (APS). These *R*^2^ values indicate a poor correlation between each of the plotted data sets. More importantly these results point to the limitations of relying solely on automated particle sizing methods.

### 3.2 Elemental analysis of each sample using EDS

EDS elemental analysis determined that the only detectable elements were Ti^4+^ and O^2−^ present in each of the seven samples in this study. Therefore, there were no contaminants of trace elements above the detection limit of 0.1% by weight. This affirmed that the received samples were the analytical grades certified by the manufacturers. It also confirmed that there were no contaminants remaining from the synthesis method. A representative set of EDS micrographs from powder population 1 [Appendix B], demonstrates the presence of only Ti^4+^ and O^2−^.

### 3.3 Crystal structure of anatase titania using powder X-ray diffraction

**Fig. 2:**
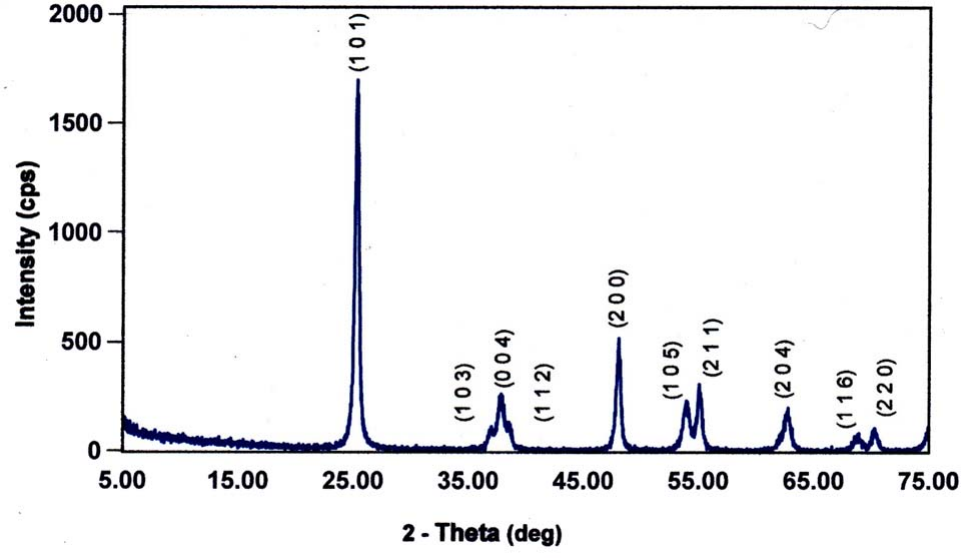
The PXRD pattern for the sample with an average diameter ≅12.69 nm. The indexed peaks fit those in the JCPDS file #00-0634-0863 (ref. 120) for synthetic anatase titania.

Each of the PXRD patterns obtained matched the JCPDS file #00-0634-0863 (ref. 120) for synthetic anatase titania, confirming the structure and composition of each powder sample. The PXRD micrograph for the powder population with an average primary particle of *d* ≅ 12.69 nm is presented in Fig. 2. The XRD micrographs for each of the other six samples are presented in Appendix C.

For a second phase in a sample to be detected in the PXRDs taken, using the equipment described in Section 2.7, there must be at least 0.2 wt% of the powder sample.^117^ From Fig. 2 any possible contaminant within the surface penetration depth of the X-ray beam was below the equipment's detection limit. Like the result in Fig. 2, the PXRD micrographs in Appendix C demonstrate only the presence of a single-phase of synthesized crystalline anatase titania. The slight count decrease in the base line [Fig. 2] from 5° 2-theta to approximately 15° 2-theta is an artifact of the equipment.^121,122^ In addition, the XRD micrographs in Appendix C support this contention, as even the particle population with a *d* = 5.31 nm has a completely flat baseline from 10° 2-theta to 75° 2-theta.

In addition, work by Samad *et al.*^123^ examined the effect of surface loading of a second oxide (*i.e.*, alumina (Al_2_O_3_)) on to a pristine surface of a primary oxide (*i.e.*, silica (SiO_2_)) on the samples PZC value. Therefore, this study can also be used to simulate the effect of surface contamination on a sample's surface. An examination of all the curves where PZC values change indicated that at/or below a surface loading of 0.2 wt%, the shift in PZC values would be less than ±0.2 pH units. This work indicates that even had there been a small amount of secondary contamination on the samples surface used in this study, its effect on the pH_PZC_ values measured would have been minimal.

### 3.4 Mass titration results

**Fig. 3:**
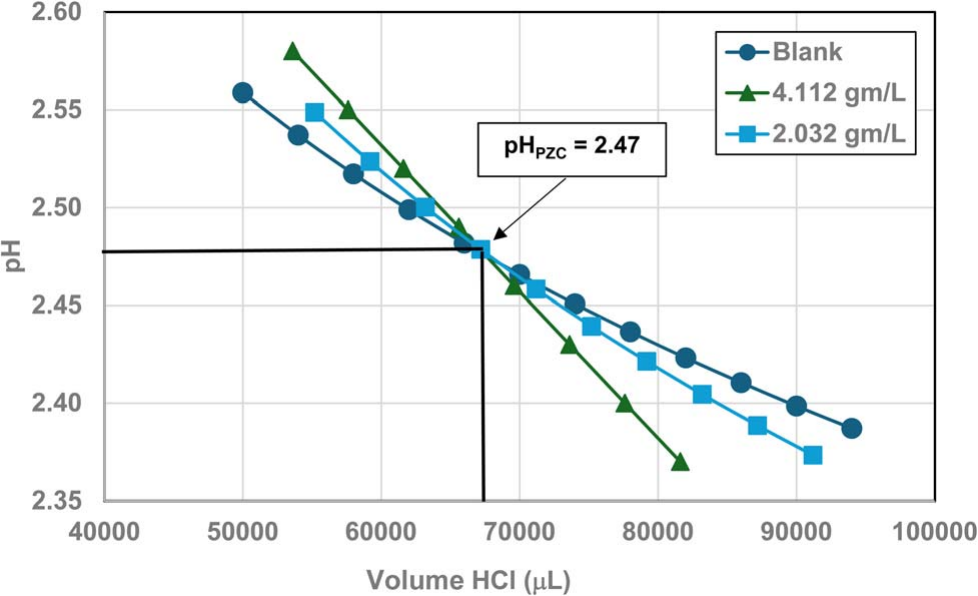
Mass titration curves of anatase titania with an average primary particle with a *d* ≅ 12.69 nm. Intersection of the three curves indicate the materials pH_PZC_ = 2.47.

Titration curves were obtained by plotting the cumulative volume of each titrant (*i.e.* 0.01 N hydrochloric acid (HCl) or 0.2 N sodium hydroxide (NaOH)) against their pH at that volume. Intersection of the three curves identified the powder populations PZC value. Fig. 3 presents the results for the powder population with an average primary particle diameter ≅12.69 nm. Appendix D presents the titration method and data for the other six other powder populations.

### 3.5 Changes in PZC values across the decreasing average primary particle diameter sizes

**Fig. 4:**
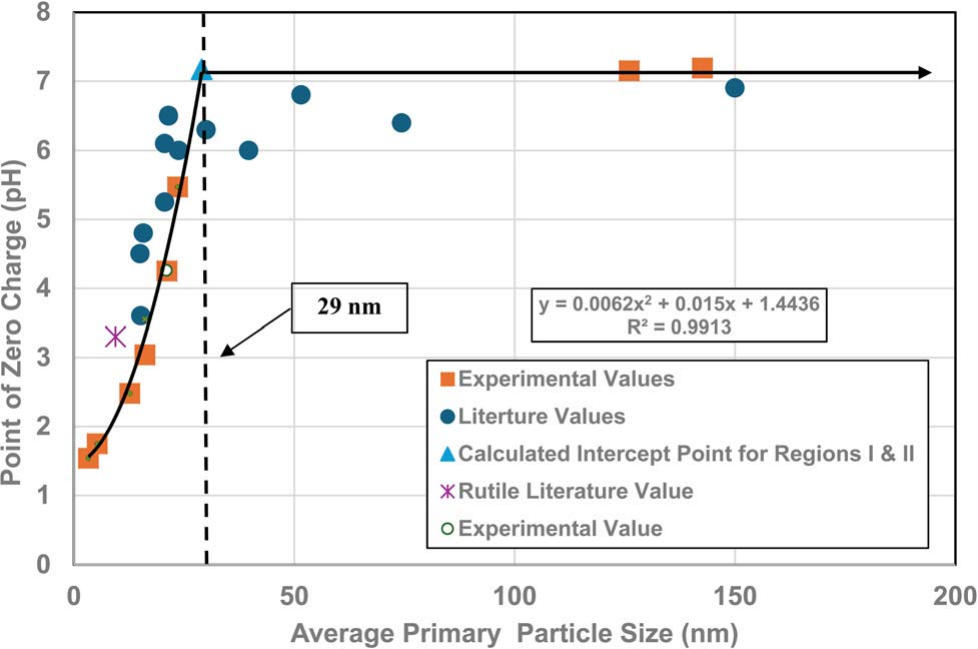
Values for both experimental and literature^124–134^ pH_PZC_ values are plotted against their normalized average primary particle diameter. The experimental point at pH_PZC_ = 4.26 was measured using 0.1 M NaOH for the electrolyte, and 0.01 M NaClO_4_ as the titrant.^134^

A plot of the normalized average primary particle sizes (*i.e.*, diameters) against its PZC value for each anatase titania sample revealed two regions [Fig. 4]. A regression curve was fitted to the experimental data in Region II. A best fit function was found to be a second order polynomial with a correlation value of *R*^2^ = 0.9921. To determine the intersection of Regions I and II. The equation for the regression curve was used to calculate the average primary particle diameter where pH_PZC_ = 7.17. The predicted intersection of the curves for Regions I and II is at *d* ≅ 29 nm, with a pH_PZC_ = 7.17.

In addition, literature pH_PZC_ values for anatase titania, which were measured within the parameters set in Section 2.0, were plotted alongside the experimental results. They confirm the shift toward lower pH_PZC_ values as the average primary particle size decreases in Region II below a *d* ≅ 29 nm. In addition, the values above *d* ≅ 29 nm, indicate pH_PZC_ values remain roughly constant (pH_PZC_ = 6.0–6.9)^124–133^ as the average primary particle diameters increase, thus supporting the findings of a Region I as indicated by the experimental values. The scatter in the literature data [Fig. 4], which also used automated particle sizing techniques, reemphasizes the limitations of these methods.

### 3.6 Correlation of PZC values and band gap

Region I demonstrates essentially no changes in the PZC values with a decreasing/increasing average primary particle diameter. The PZC values of each particle population are pH = 7.15 (*d* ≅ 126 nm) and PZC = 7.19 (*d* ≅ 142 nm), are well within the calibration error (pH = ± 0.2) of the equipment (HI 2214 pH/ORP meter). The difference between the two powder populations diameters is ∼16 nm. In Region II [Fig. 4] a change of this magnitude in the average particle diameter of two powder populations would result in a shift of its PZC value by approximately 3.5 pH units. This demonstrates that the underlying physical parameters responsible for changing PZC values in Region II does not occur above the average primary particle sizes greater than *d* ≅ 29 nm in Region I.

The results in Fig. 4 also indicate that the experimental procedure used (Section 2) succeeded in minimizing the effects of all the other factors cited. Fig. 4 suggests that there is an unidentified physical parameter responsible for the decrease in PZC values below the normalized average primary particle size of *d* ≅ 29 nm for anatase titania. This parameter would need to be found at the surface, as pH_PZC_ is purely a surface measurement.

The PXRD patterns [Fig. 21, Appendix C] of each powder population confirmed that the structures are anatase titania. As the bulk/surface structures of this material are known to be identical^9,10^ this confirms the structure at the solid/liquid interface is anatase titania. Based on Eh-pH diagrams, the dominant species TiO_2_ is stable from a pH = 1 to pH = 14.^35^ This eliminated any thermodynamic driving force that would change the bulk/surface structure's oxidation state and/or coordination number, as pH_PZC_ values in Region II decrease.

One of the physical parameters known to change with decreasing average primary particle size below a given diameter, is anatase titania's indirect band gap. Lin *et al.*^111^ demonstrated that this is the result of increasing surface bond lengths. Their work demonstrated that the average diameter of anatase titania at which the indirect band gap values begin decreasing is at *d* ≅ 28 nm, within 1 nm of the projected intercept point (*d* ≅ 29 nm) of Regions I and II [Fig. 4]. This diameter is effectively identical to the calculated value at which pH_PZC_ values start decreasing. That made it possible to obtain values for the indirect band gap of anatase titania from the literature. The protocol used to choose the band gap literature values are as follows:

(1) The parameters used to determine the method for minimizing the factors affecting pH_PZC_ values were applied to the measured indirect band gap values from the literature [Section 2].

(2) The literature used presented all the raw data used to obtain their results (*i.e.*, transmission electron micrographs), specific surface area measurements (BET), band gap spectra, powder X-ray diffraction, high resolution transmission electron microscope, TEM, *etc.* The results presented were then reconfirmed using the author's raw data.

(3) The particle populations, whose band gap values were used, possessed a cubic to spherical morphology, thereby matching the shapes present in powder populations measured [Fig. 16, Appendix B]. This avoided variations in the data used due to differing particle morphologies, that is known to affect indirect band gap values.^135,136^

(4) Band gap measurements were made at ambient temperature and pressure to match the experimental conditions under which each sample population pH_PZC_ was measured. This is because temperature and pressure have been demonstrated to affect band gap values [ref. 137, Appendix A].

(5) The samples were not pretreated as this is known to affect the material's indirect band gap.^138^

**Fig. 5:**
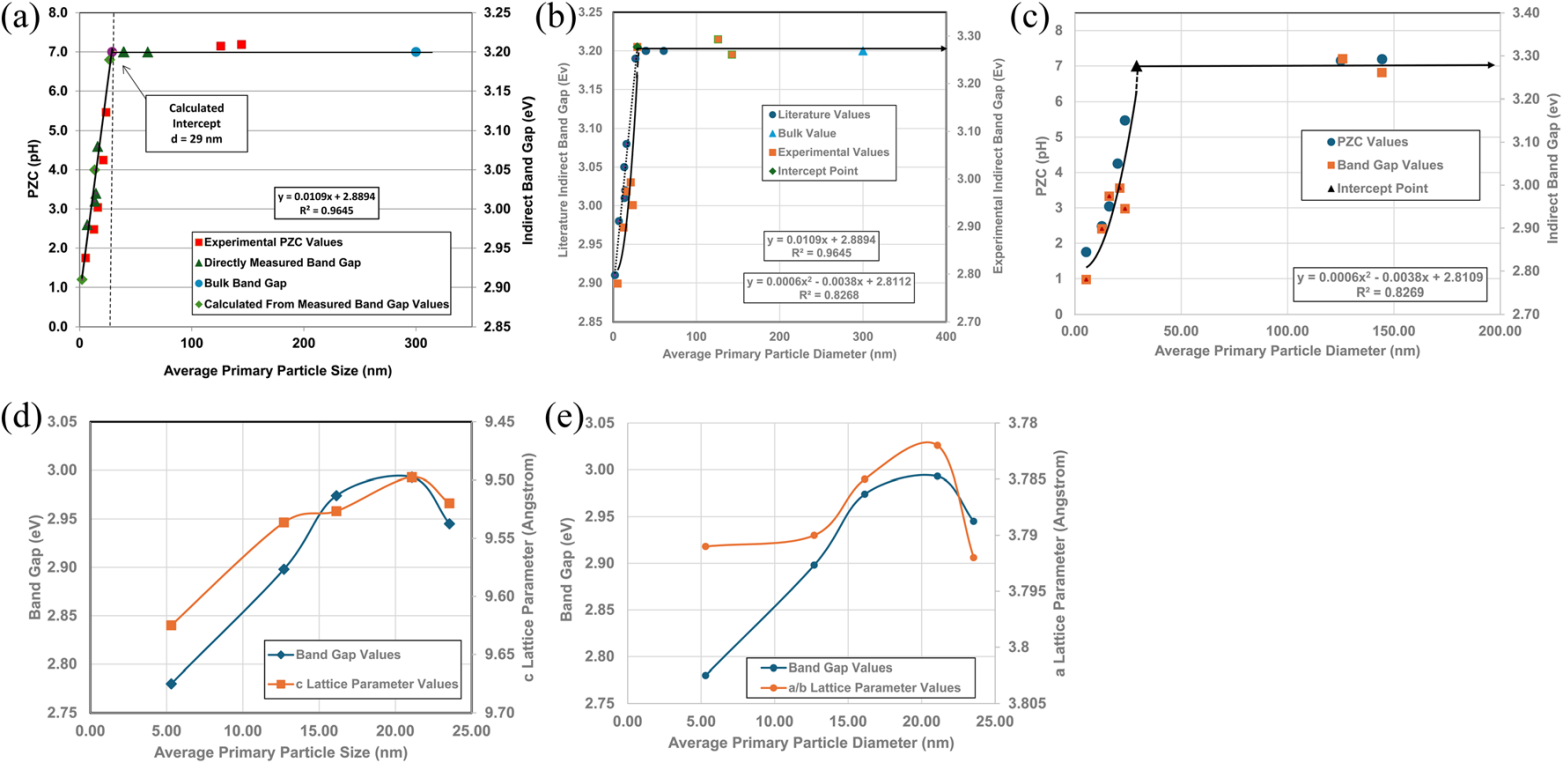
(a) A visual correlation of experimental PZC and literature indirect band gap values^137–142^ against their average primary particle diameter. The equation calculates band gap values. (b) Visual correlation between the literature and experimental indirect band gap values. The literature values gave a best fit curve for a linear function, with a correlation value *R*^2^ = 0.9645.^137–142^ Experimental values gave a best fit curve for a polynomial function with an *R*^2^ = 0.8268. (c) A plot of both the experimental PZC and indirect band gap values against their average primary particle size. The equation calculates band gap values. (d) A visual correlation between band gap and *c* lattice parameter values plotted against their average primary particle diameter. The *c* lattice parameter values have been reversed to visually correlate the two curves more easily. (e) A visual correlation between band gap and *a*/*b* lattice parameter values plotted against their average primary particle diameter. The *a*/*b* lattice parameter values have been reversed to visually correlate the two curves more easily.

A plot of multiple literature indirect band gap values^139–145^ and experimental pH_PZC_ values against their average primary particle diameter demonstrated a clear visual correlation between the two parameters [Fig. 5a]. A regression curve fit of the indirect band gap values in Region II resulted in a correlation value of *R*^2^ = 0.9645 with an intercept value between the two Regions of *d* ≅ 29 nm. In Region I there is essentially no change in the indirect band gap and pH_PZC_ values. As the average primary particle size decreases in Region II, the indirect band gap values (*i.e.* increasing surface bond lengths) and pH_PZC_ values demonstrate an almost identical shift toward lower values along the fitted regression curve. These initial results demonstrate that there is a strong visual correlation between the indirect band gap and all the experimental pH_PZC_ values.

To confirm that the indirect band gaps for each sample followed the pattern found in Fig. 5a, samples from each powder population were sent to EAG Laboratories in the Netherlands. Both the experimental and literature indirect band gaps were then plotted against their average primary particle diameter on the same graph. The results of these plots are presented in Fig. 5b. Due to the slight offset between the two sets, their band gaps were plotted on different *y* axis. This offset was most likely due to sample preparation [Appendix E]. Both curves consist of a Region I and Region II which mirror each other.

The best fit function for the experimental band gap value in Region II was found to be a second order polynomial with an *R*^2^ = 0.8268. The intercept point band gap values at *d* = 29 nm are the average of the two points in Region I (eV = 3.277) and is included in the fitted curve. They demonstrate that the results also follow the same pattern found in Fig. 5a, where a strong visual correlation between the experimental and literature values is observed [Fig. 5b].

Fig. 5c presents a plot of experimental values for each sample's PZC and indirect band gap against their average primary particle diameter. The two plots allow for a visual correlation of the data. The intercept point (*d* = 29 nm) was used for the fitted curve of the band gap values and is the average of the measured bulk band gap values [Appendix E] in Region I. The fitted trend line for the experimental indirect band gap values, including the projected intercept point, has an *R*^2^ = 0.8269.

Fig. 5a and c demonstrate that surface bonds (*i.e.*, band gaps) begin to lengthen below an average primary particle diameter of *d* ≅ 29 nm. To determine how the bonds in the lattice expand or contract, lattice parameters *a*/*b*, and *c* were measured from their PXRD patterns (Fig. 21, Appendix C) using the Rigaku PDXL 1.8.0.3 software. These values were also used to determine if surface roughness might have affected the measured pH_PZC_ values.

Band gap values from *d* = 5.31 nm to *d* = 21.08 nm continually increase. Above this last particle size, the band gap then decreases significantly from eV = 2.993 to eV = 2.945 at the next particle size *d* = 23.54 nm. Fig. 5d and e illustrate the underlying cause for the shift downward of the band gap from particle sizes *d* = 21.08 nm to *d* = 23.54 nm. In Fig. 5d the curves for experimental band gaps and *c* lattice parameters demonstrate a strong visual correlation between *d* = 5.31 nm to *d* = 23.54 nm. This indicates a close paralleling of the expansion and contraction of the surface *c* lattice parameter bonds.

In Fig. 5e the *a*/*b* lattice parameter surface bonds between particle sizes *d* = 12.69 nm to 23.54 nm parallel the experimental band gap values. Below a particle size of *d* = 12.69 nm the *a*/*b* lattice parameters remain essentially constant, while the band gap value continues to decrease. This suggests that the *c* lattice parameters may play a more significant role in surface bond length (*i.e.*, band gap) expansion than those for the *a*/*b* lattice parameters at the smallest average primary particle diameter.

Surface band gaps have been shown to correlate directly with surface roughness.^146^ In this case though, Fig. 5d and e demonstrate that the band gap values correlate directly with changes to the materials surface structure (*i.e.* bond lengths). Therefore, it was determined that there was insufficient surface roughness present on each of the particle populations used to affect the materials band gap values. Nor was it responsible for the band gap decrease from *d* = 21.08 nm to *d* = 23.54 nm.

### 3.7 Correlation of PZC values and lattice parameter ratios (*c*/*a*)

**Fig. 6:**
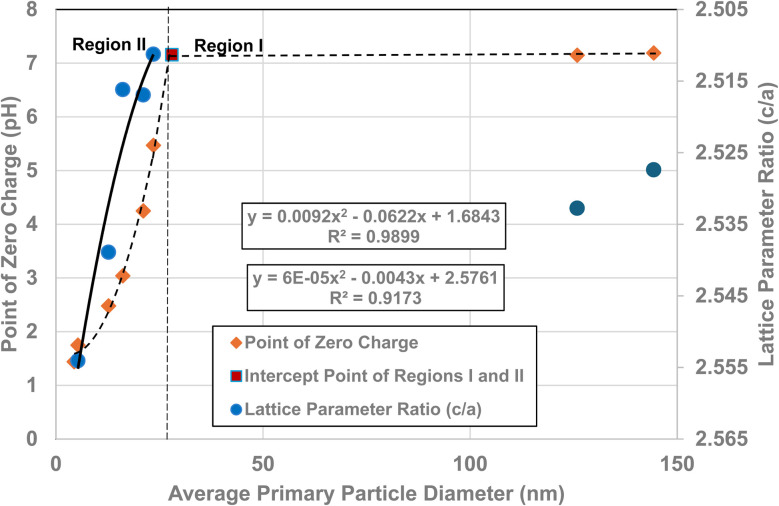
A visual correlation between the experimental lattice parameter ratios (*c*/*a*) and experimental PZC values against their average primary particle diameters. The lattice parameter ratios (*c*/*a*) values are reversed to provide a clearer visual correlation with their PZC values.

Fig. 5a and c demonstrate that surface bonds (*i.e.*, band gap) begin to lengthen below an average primary particle diameter of *d* ≅ 29 nm. Using the measured lattice parameters the lattice parameter ratio (*c*/*a*) was calculated for each sample. These results were then plotted against their average primary particle diameters [Fig. 6]. Lattice parameter ratio data in Region II were found to have a best fit function of a 2nd order polynomial with an *R*^2^ = 0.9173. The fitted curve for the experimental pH_PZC_ values gave an *R*^2^ = 0.9899.

The curve in Fig. 6 demonstrates that as the average primary particle diameter decreases, the lattice parameter ratios (*c*/*a*) indicate that the *c* lattice parameter expands significantly with respect to the *a*/*b* lattice parameters. An examination of the change in the individual lattice parameter lengths was made as particle size decreased from a diameter of 23.54 nm to 5.31 nm. The *a*/*b* lattice parameters contracted slightly by ∼– 0.24%, while the *c* lattice parameters expanded by ∼+ 1.04%. The lattice parameter ratio (*c*/*a*) in Region I remains approximately the same, with a difference of only ∼8.4 × 10^−4^ nm (0.21%). These results suggest that the lattice parameter ratios (*c*/*a*) in Region I (*i.e.*, the bulk structure) change very little [Fig. 5a–c], and by extension surface bond lengths remain constant.

### 3.8 Quantitative correlation of PZC values, lattice parameter ratios (*c*/*a*) and band gap

Fig. 4, 5c and 6 demonstrate strong visual correlations between the experimental pH_PZC_, band gap and lattice parameter ratio (*c*/*a*) values. To confirm these correlations, it was necessary to demonstrate a quantitative relationship between the two structural parameters and pH_PZC_. This was achieved by using the equations in Fig. 4, 5b (experimental calues), and 6, fitted to the portion of the curve in Region II for pH_PZC_, band gap, and lattice parameter ratio (*c*/*a*) values. This was possible, as all three regression curves have *R*^2^ values greater than 0.82, indicating a strong correlation between the data sets plotted in these figures. To achieve this, values for pH_PZC_, band gap, and lattice parameter ratio (*c*/*a*) were generated for diameters from 6 nm to 24 nm, in increments of 2 nm. This range was chosen as there is physical data to compare with the calculated values. As the generated values were for the same particle diameter, the factors could then be plotted directly against each other.

**Fig. 7:**
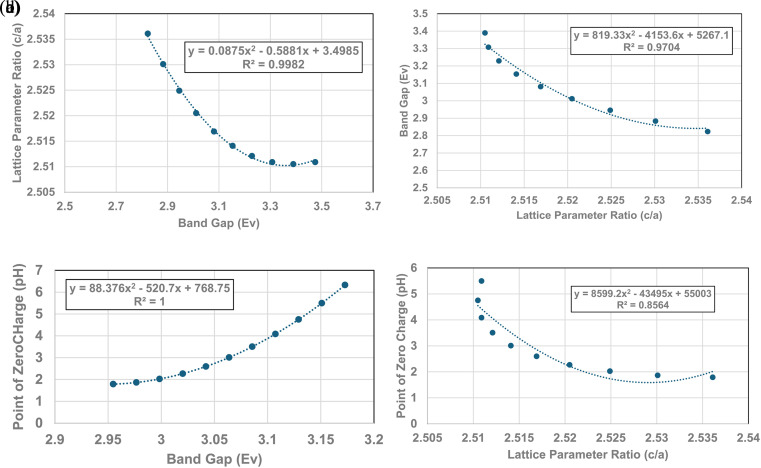
(a) Plot of the calculated band gap values against the lattice parameter ratio (*c*/*a*) in Region II. (b) Plot of the calculated lattice parameter ratio (*c*/*a*) against its band gap (eV) in Region II. (c) Quantitative correlation between the calculated surface band gap (*i.e.*, bond lengths) and their PZC values in Region II. (d) Quantitative correlation between calculated lattice parameter ratio (*c*/*a*) and point of zero charge values in Region II.

To determine the correlation between the two structure parameters, band gap (eV) (*x* axis: independent variable) and lattice parameter ratio (*c*/*a*) (*y* axis: dependent variable), were plotted together in Fig. 7a. A regression curve was then fitted to the calculated values. A 2nd order polynomial was found to be the best fit function between these two parameters, with an *R*^2^ = 0.9969. When the plot of the two data sets was reversed (*i.e.*, lattice parameter ratio (*c*/*a*) → *x* axis, band gap → *y* axis), making the lattice parameter ratio (*c*/*a*) the independent variable [Fig. 7b], the *R*^2^ value for the regression curve decreased to 0.9677. The higher correlation factor in Fig. 7a indicates that the lattice parameter ratio (*c*/*a*) is the dependent variable in the relationship. Therefore, the expansion and/or contraction of the band gap (*i.e.* surface bond lengths) are the independent factor responsible for the change in lattice parameter ratio (*c*/*a*) values.

The calculated values for the material's band gap and lattice parameter ratio (*c*/*a*) were then plotted as the independent variable against the materials calculated pH_PZC_ values as the dependent variable. The curves are presented in Fig. 7c and d. Regressions were fitted to both curves in Fig. 7c. A 2nd order polynomial was found to be a best fit function for both curves. Based on the correlation values in Fig. 7c (*R*^2^ = 1.0) and 7d (*R*^2^ = 0.8564), the higher correlation factor in Fig. 7c indicates that materials band gap (*i.e.*, surface bond lengths) value is the independent factor responsible for changes in the materials PZC values.

## 4 Discussion

Fig. 5a and c demonstrate that in Region I, pH_PZC_ does not change as the particle size increases/decreases, nor do their band gap (*i.e.*, surface bond length) values. Based on the Nernst Equation [eqn (8)]^147^ the potential difference (*ψ*_0_) goes to zero at the aqueous/solid interface when the specific surface charge (coulombs per m^2^) and the concentration of counter ions (mol L^−1^) in solution at the surface (pH_PZC_) are equal. Under this condition the surface potential (*Ψ*_0_) equals zero so that it can no longer draw counter ions out of solution to adsorb on to the surface.8
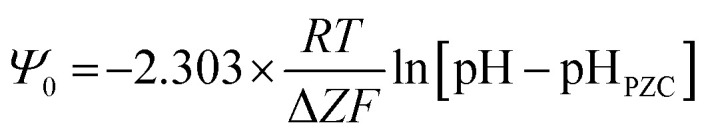
where *R* = universal gas constant (8.314 J K^−1^ mol^−1^), *T* = temperature (K), Δ*Z* = change in the charge of surface groups (+1 or −1 in the case of protonation/deprotonation), *F* = Faraday's constant (96 485 C mol^−1^), pH_Solution_ = concentration of counter ions in solution (mol L^−1^) both at and away from the surface, pH_PZC Solution_ = concentration of the solution counter ions (mol L^−1^) at and away from the surface which results in zero potential at the aqueous/solid interface.

Based on eqn (8), the material's specific surface charge (coulombs per m^2^) for particles in Region I does not change since each sample's pH_PZC_ remains constant (pH_PZC_ ≅ 7.17). In Region II, the decrease in pH_PZC_ values indicates that the property responsible for the increase in the materials positive specific surface charge is changing. This is evident from the increase in the specific concentration of negative counter ions at the surface (pH_PZC_) needed to take the aqueous/solid interface potential to zero. Based on the quantitative correlations in Fig. 7c this property appears to be controlled by changes in the materials surface band gap (*i.e.*, surface bond lengths).

A possible explanation for changes in anatase titania's specific surface charge might be due to the material's ionic/covalent bonds. This type of bond possesses an asymmetric electron charge distribution,^146^ which creates surface dipoles.^149^ The polarity of an electric dipole is quantified by its electric dipole moment (*µ* = coulombmeters) [eqn (9)]:^148^

9 *µ* = *q* × *e* × *d*

where *q* = 1 [indicating a complete separation of the unit charges], *e* = 1.602 × 10^−19^ Coulombs, *d* = distance (10^−10^ m).

Work by Yan *et al.*,^150^ using molecular dynamic simulation (MD) demonstrated a linear relationship between anatase titania's increasing surface area (nm^2^) and its electric dipole moment. Their model determined values for surface areas between 12.8 nm^2^ (*d* = 120.8 nm) to 78.5 nm^2^ (*d* = 19.6 nm). The electric dipole moments increased linearly from 27.5 Deby (D) to 182.1 D with increasing surface area.

Eqn (10) might explain possible changes to the overall ionic character (*I*_%_) of a material's surface bonds^148^ using the surface and bulk structure bond dipole moments. In Region I the average surface bonds are approximately 3% to 4% shorter than those within the bulk structure.^151^ Therefore, a value for the observed dipole moment (*µ*_obs_) can be determined from the shorter surface bonds.^149,150^ The theoretical dipole moment (*µ*_100%_) could then be calculated from the longest possible average bond length, which is located within the materials bulk structure.^146^10
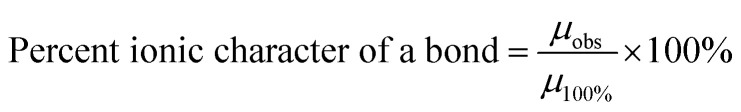


As the particle size decreases in Region II, surface bonds expand (Fig. 5a–c) resulting in the observed dipole moment of the surface bonds increasing [eqn (9)]. This results in an increase in the ionic character of the surface bonds [eqn (10)]. At the particle size where surface bonds become equal with those within the bulk (Region II) suggests they might become fully ionic (*i.e.*, *I*_%_ = 100%). This might also indicate that the bulk M^2+^–O^2−^ bonds are already fully ionic.

Evidence available in the literature supports this shift in bond ionic character as particles size decreases into the nanoscale region. Pauling's^152^ work on electronegativity is based on the concept that each atom has a single set value. He used the bond energy of atoms to determine their electronegative value, which is typically measured using a known mass of a material at its melting temperature. His work though, did not account for the particle size effect on the melting point of materials. As the average primary particle size decreases into the nanoscale region its melting point is significantly depressed.^153^ Some examples of this are gold (Au),^154^ silver (Ag),^155^ tin (Sn),^156^ copper (Cu) and cobalt (Co)^157^ particles. The curves for these examples mirror the pH_PZC_ results in Regions I and II in Fig. 4, 5a, c and 6.

Work by Gibbs *et al.*^158^ examined the question of changing electronegative (*χ*) values due to the increasing coordination number in different elements. As the coordination number of an anion about a cation increases the ionic radius of the cation becomes larger. This results in an increase in the bond lengths between the anions and cations in the structure.^110^ Gibbs *et al.*^158^ also determined that as a bond lengthens the electron density along its bond path decreases. This in turn decreases the electronegative value of the cation.

One of the examples they present is for the silicon (Si) atom. Silicon, with a coordination number (CN.) of 4, has a *χ* = 1.81, the same as found in Pauling's electronegative table.^152^ As the coordination number of Si increases to 6, then 8, *χ* = 1.70 and 1.46 respectively. Calculating the ionic character^150–152^ for these Si^4+^–O^2−^ bonds [eqn (11)] results in a shift in the values from *I*_%_ = 51.03% (CN: 4), to *I*_%_ = 55.51% (CN: 6), and finally *I*_%_ = 64.67% (CN: 8).11
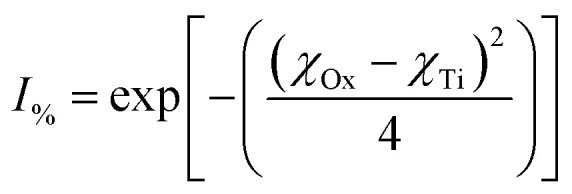
where *χ*_Ox_ = 3.5, *χ*_Si_ = 1.81, 1.70, 1.40.

In total, the Si^4+^–O^2−^ bond ionicity increases by 13.64% with increasing bond length. Therefore, it is reasonable to conclude that expanding bond lengths directly affects the overall ionic character possibly due to its increased surface dipole moments.

Using this information, a model was developed to explain the stability of pH_PZC_ values in Region I and their decreasing values in Region II based on structural surface changes. In Region I the surface structure is suggested by Livey and Murray's^159^ work and supported by density functional modeling of the anatase titania surface by Oliver *et al.*^160^ Both determined that surface atoms arrange themselves to reduce surface energy to their lowest state. This is achieved by the surface cations (*i.e.*, Ti^4+^) retracting downward toward the bulk, while the anions (O^2−^) pull up and over the cations, partially covering them. An examination of the structure factors in Region I finds that band gap values in Fig. 5a–c and lattice parameter ratios (*c*/*a*) [Fig. 6] remain essentially constant. Therefore, the surface structure (*i.e.*, bond lengths) remain little changed with respect to the solid/aqueous interface above an average primary particle size of *d* ≅ 29 nm.

When metal/oxygen atoms bond the electrons lost from the metal atoms are fully transferred to the oxygen.^110^ At the surface, in Region I, bond lengths for the six coordinated metal atoms are approximately 3–4% shorter than within the bulk structure.^151^ Therefore, based on Coulomb's Law^161^ [eqn (12)] the attractive electrostatic force (*F*) between the metal (+) and oxygen (−) atoms at the surface in Region I would be stronger than within the bulk structure. This might have resulted in the transferred electron density around the surface oxygen bowing back toward the positively charged metal atoms. The distorted portion of this transferred electron density might have then partially shielded some of the positive charge on the surface metal atoms, which are seated lower than the surface oxygen. This might have resulted in reduction of the overall global fraction of the positively charged surface which is exposed at the solid/aqueous interface.^159,160^12
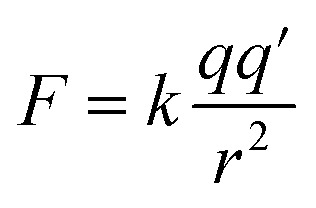
where *F* = force (N), *k* = proportionality constant (8.98755 × 10^9^ N m^2^ C^−2^), *q*, *q*′ = point charges (Coulombs), *r* = distance between the point charges (nm).

As the surface bond lengths expanded between the metal and oxygen atoms in Region II (Fig. 5a–c), electrostatic attractive force between the metal atoms and the transferred electron density about the oxygen [eqn (12)] would have decreased.^161^ This might have resulted in the distorted electron density, which had bowed toward the metal atoms in Region I, retracting back toward the oxygen in Region II. This correlates with Gibbs *et al.*'s^158^ findings that as bond lengths increase, electron density along the bond path decreases. With the loss of the electron density shielding part of the metal surface atoms, and their movement upward toward the surface, the effect would be to increase the global fraction of the surface possessing a positive charge. This is supported by the decreasing pH_PZC_ values in Region II, which indicate that the concentration of negatively charged counter ions being adsorbed at the surface, needed to shift surface potential to zero, is increasing [eqn (8)] due to a higher specific (*i.e.* global) surface area with a positive charge.

The increase in surface bond lengths might also account for the contraction of the *a*/*b* lattice parameters. As the distorted electron density retracted back toward the oxygen atoms in Region II, the electron density might have become more evenly distributed about the surface oxygen atoms, resulting in the anions becoming more spherical. It might have then resulted in a decrease in their ionic radius, thereby reducing the volume occupied by each oxygen atom in the lattice. This might account for the small contraction (∼0.24%) of the *a*/*b* lattice parameters.

## 5 Conclusions

The results in this work demonstrate that a protocol to selectively minimize the effect of all the factors affecting a metal oxide's pH_PZC_ has been developed. The findings obtained using this method allowed identification of the surface structure factor and property affecting a metal-oxide/hydroxide's point of zero charge. This was achieved by first demonstrating a direct visual correlation of the pH_PZC_ values with the materials band gap (*i.e.*, surface bonds) [Fig. 5a and c], and lattice parameter ratio (*c*/*a*) [Fig. 6]. The quantitative correlations established [Fig. 7a–d] demonstrate changes in the materials surface band gap (*i.e.*, surface bond length) is the independent structural parameter responsible for the shift in both the lattice parameter ratio (*c*/*a*) and pH_PZC_ values in Region II. The final correlation found between surface bond expansion [Fig. 5a and c] and pH_PZC_ was the increase in the surface bond's percent ionic content [eqn (9) & (10)] in Region II. These findings also demonstrate that particle size and morphology, while affecting those surface structure factors and properties responsible for the change in pH_PZC_ values, are not the underlying cause responsible for this change.

## Data availability

All data is available on request.

## Conflicts of interest

There are no conflicts to declare.

## Appendices

### A Appendix A

#### A.1 Approach and method used to calculate the normalized spherical diameters for goethite

Work by Kosmulski *et al.*^44^ determined that synthetic goethite crystals with a specific surface area (SSA) below approximately 60 m^2^ g^−1^, unless specifically indicated, have short lengths, resulting in morphologies that are approximately cubic to rectangular. This allows the use of the material's SSA and eqn (13) (ref. 41) to obtain an average spherical diameter. Goethite crystals with an SSA above 60 m^2^ g^−1^ typically possess an acicular (*i.e.*, needle like) morphology. Therefore, two sets of data for synthetic goethite were chosen, each representative of one of these morphologies. When choosing a data set from the literature, samples which had been freeze dried,^45^ ground and/or sonicated^46^ were omitted as these processes are known to damage and/or decrease crystallite sizes.13
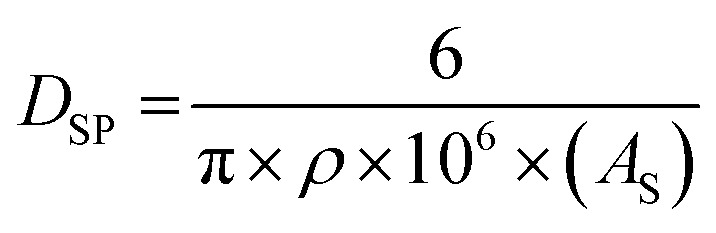
where *D*_SP_ = diameter for a sphere (nm), *ρ* = density (g cm^−3^), *A*_S_ = specific surface area (m^2^ g^−1^), correction factor = 1 × 10^6^ (cm^3^ m^−1^).

Group 1 possesses SSA's below 60 m^2^ g^−1^, with none of the source literature specifying dimensions for an acicular crystal.^47–55^ This indicated that the average particle morphology was roughly cubic to rectangular. Group 2 data sets, with SSAs over 60 m^2^ g^−1^, did specify that the particles possessed an acicular (needle like) morphology.^56–59^ All the data sets used provided PZC, and specific surface area. Data sets possessing acicular morphologies also provided the dimensions (*i.e.*, length, width, depth) for the average primary particle size of that population.

The first step was to determine what happened when the average primary particle diameter was determined using only one measurement method for both data sets. This was accomplished using eqn (13), each data set's SSA and the density (*ρ* = 4.28 g cm^3^) for goethite.^42^ Then the calculated average primary particle diameter for each data set was plotted against its PZC value. The results are presented in Fig. 1a.

An examination of the results demonstrates that PZC values for Group 1 (cubic to rectangular particles) remains constant above an average spherical primary particle diameter of approximately 65 nm. Below a *d* ≅ 65 nm, PZC values then decrease almost linearly. Group 2 (acicular particles) demonstrates little apparent change in the material's PZC value regardless of the particle diameter.

From the results in Fig. 1a, there appears to be no correlation between the two groups even though they all possess the same phase. The only difference between them is their morphology.

The cause of this discrepancy is most likely due to eqn (13), as the spherical/cubic/rectangular particle diameter is inversely related to its SSA. This geometric relationship though does not hold for the acicular particles in Group 2. These differences may be why morphology^2^ was identified as affecting PZC values.

The next step was to calculate diameters for the acicular particles had they possessed spherical to cubic morphologies with the same volume. This was accomplished by first determining the volume (*V*_AP_) of the average acicular particle size for its population from the dimensions provided in each paper.^56–59^ The average acicular volume was then set equal to the equation for a sphere (*V*_S_ = 4/3 × π × *r*^3^)^60^ and rearranged to obtain the particle's equivalent spherical radius (*R*_ES_) had it possessed that morphology [eqn (14)]. Placing the equivalent spherical radius into eqn (15) gives the equivalent spherical diameter (*D*_ES_) for the acicular particle. Therefore eqn (14) and (15) effectively normalized the morphology of the particles in Groups 2 with Group 1.14
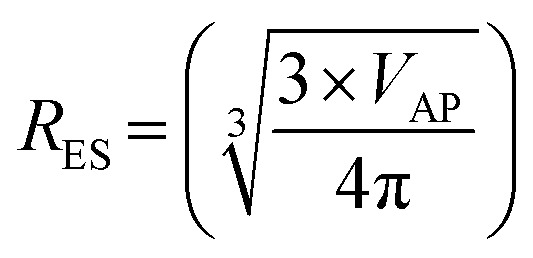

15 *D*_ES_ = 2 × *R*_ES_

When the *D*_ES_ values were plotted against their PZC values alongside the data sets from Group 1 [Fig. 1b] a single curve emerged. The fitted regression curve, with a correlation factor (*R*^2^ = 0.9058) indicates that the PZC values, the spherical to cubic particle diameters (*D*_SP_) and the normalized/equivalent acicular diameters (*D*_ES_) sets, at and below approximately 65 nm are strongly correlated.

#### A.2 Multiple examples of average primary particle size *vs.* PZC from the literature (Fig. 8–12, Table 2)

**Fig. 8:**
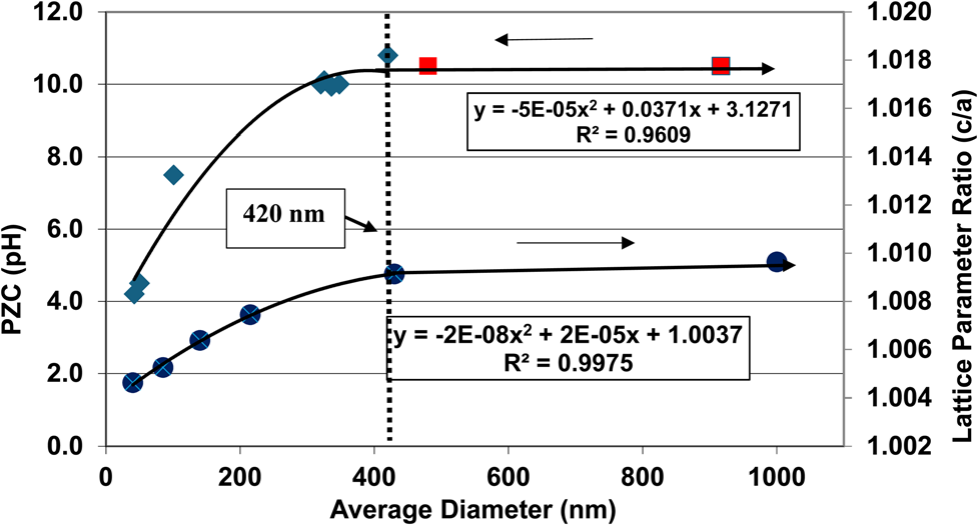
Plots of the average primary particle size, PZC values and their lattice parameter ratio (*c*/*a*) for BaTiO_3_.^61–72^ The method presented in Section A.1 was used to obtain the average diameter of each data set. The two sets of data were plotted together so a visual correlation could be obtained.

**Fig. 9:**
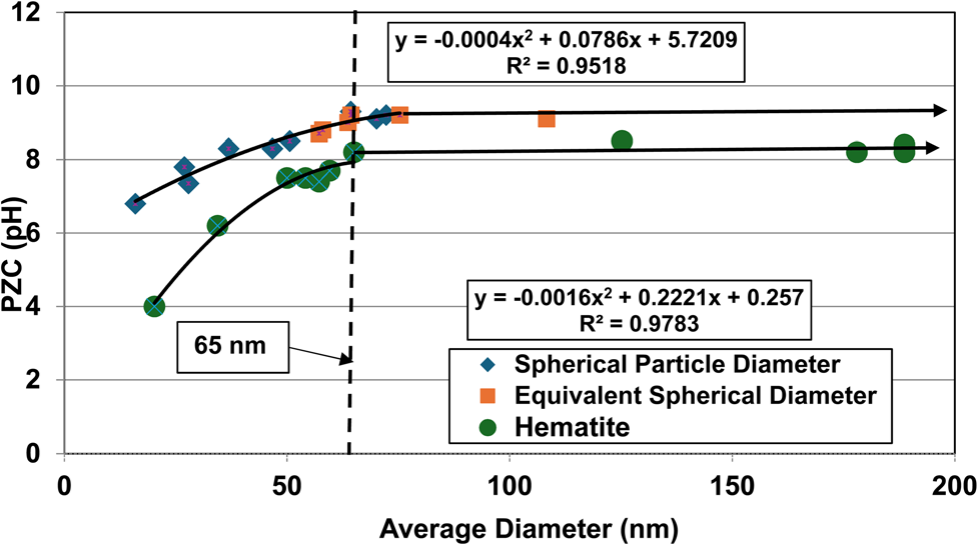
Plots of the average primary particle size against their PZC values for goethite (upper curve)^47–59^ and hematite (lower curve)^86–95^ were plotted together so the results could be visually correlated. The method presented in Section A.1 was used to obtain the average diameter of each data set.

**Fig. 10:**
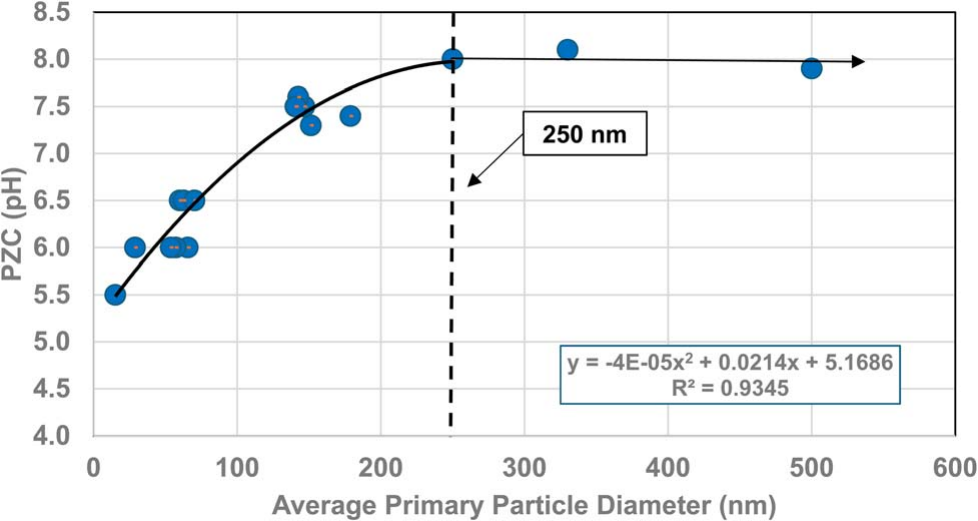
A plot of the average primary particle size and PZC values for 3% doped tetragonal ZrO_2_.^96–109^ The method presented in Section A.1 was used to obtain the average diameter of each data set.

**Fig. 11:**
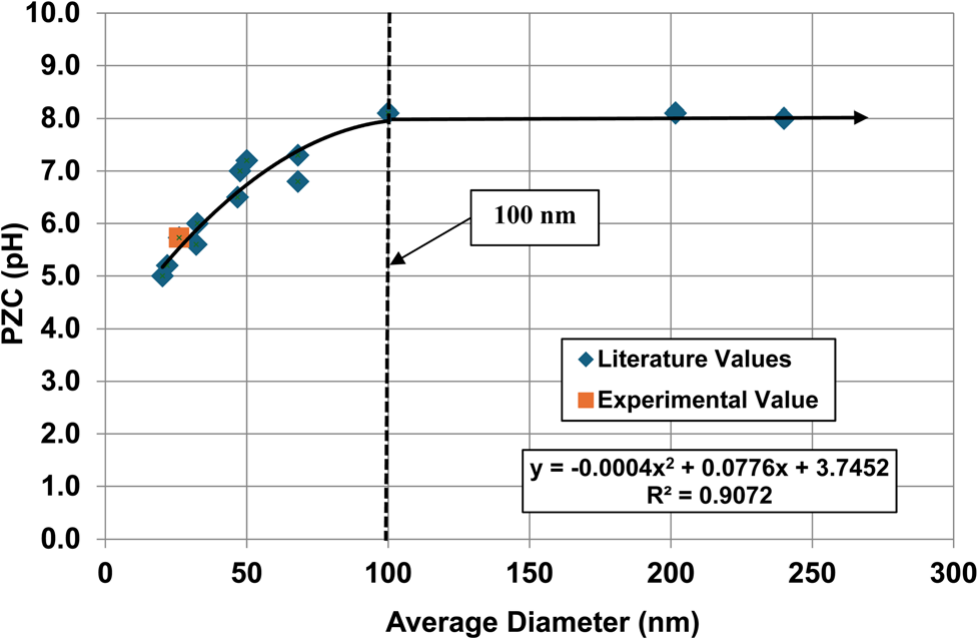
A plot of the average primary particle size against their PZC values for monoclinic zirconia (ZrO_2_).^73–85^ The method presented in Section A.1 was used to obtain the average diameter of each data set. The experimental point was measured in 0.1 M NaClO_4_ electrolyte using 0.01 M NaOH as the titrant.^134^

**Fig. 12:**
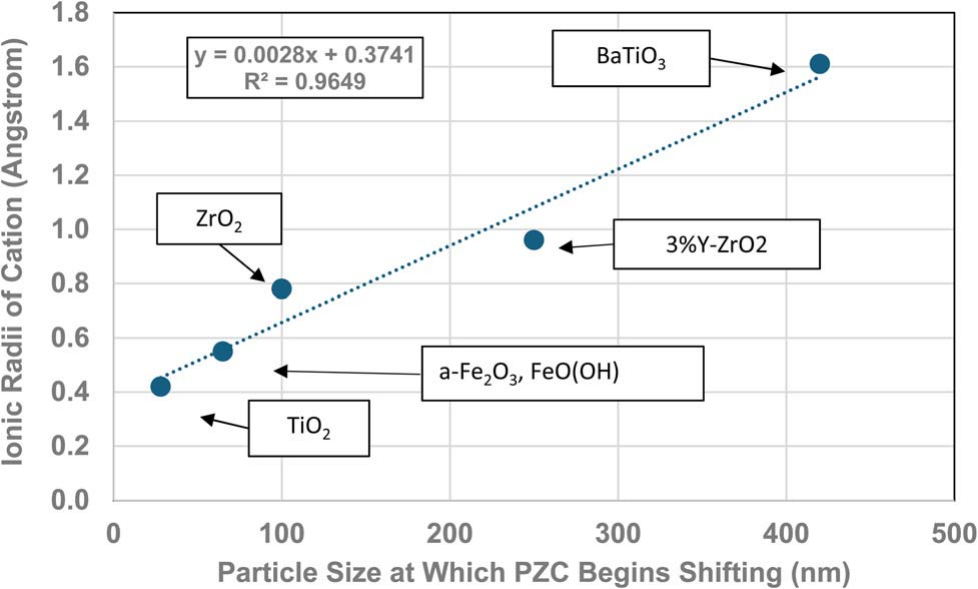
The effect of the largest cation ionic radius on the particle size at which the metal-oxide PZC values begin to decrease.^110^

**Table 2:** Values for largest cation in each metal oxide, their ionic radius, oxidation state and coordination value. The column particle size refers to the diameter at which pH_PZC_ values began decreasing^110^

Formula	Particle size	Coord. no.	Oxidation #	Ionic radius (Angstrom)	Largest cation
ZrO_2_	100	7	4	0.78	Zr
TiO_2_	28	6	4	0.42	Ti
BaTiO_3_	420	12	2	1.61	Ba
3% Y-ZrO_2_	250	7	3	0.96	Y
FeO(OH)	65	6	3	0.55	Fe
α-Fe_2_O_3_	65	6	3	0.55	Fe

### B Appendix B

#### B.1 TEM micrographs of each anatase titania sample (Fig. 13–20).

**Fig. 13:**
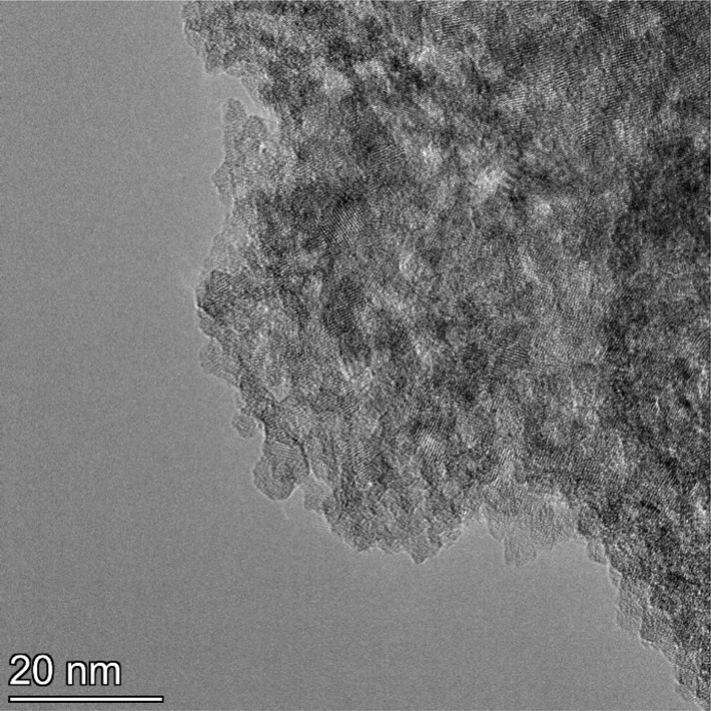
Powder population 1 with an average primary particle diameter of 5.307 nm ± 0.527 nm.

**Fig. 14:**
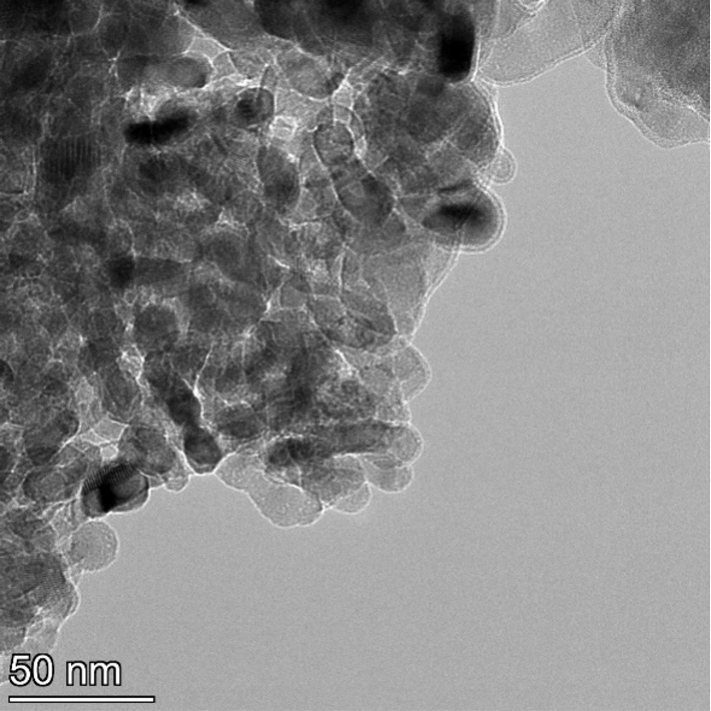
Powder population 2 with an average primary particle diameter of 12.689 nm ± 2.400 nm.

**Fig. 15:**
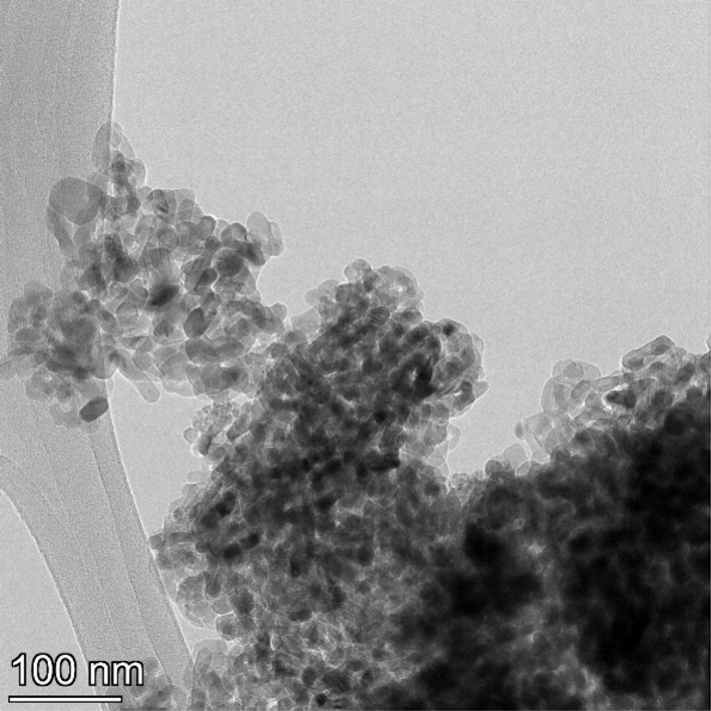
Powder population 3 with an average primary particle diameter of 16.131 nm ± 3.958 nm.

**Fig. 16:**
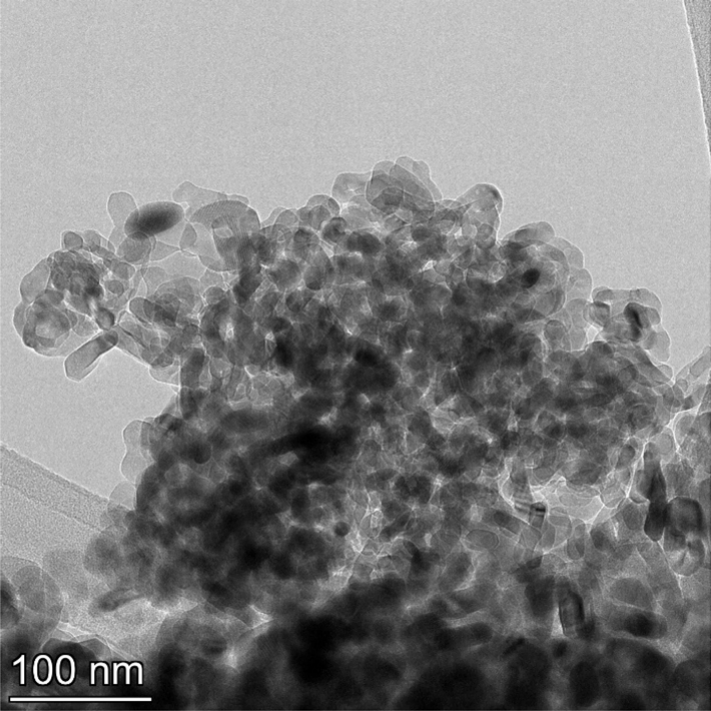
Powder population 4 with an average primary particle diameter of 21.108 nm ± 4.059 nm.

**Fig. 17:**
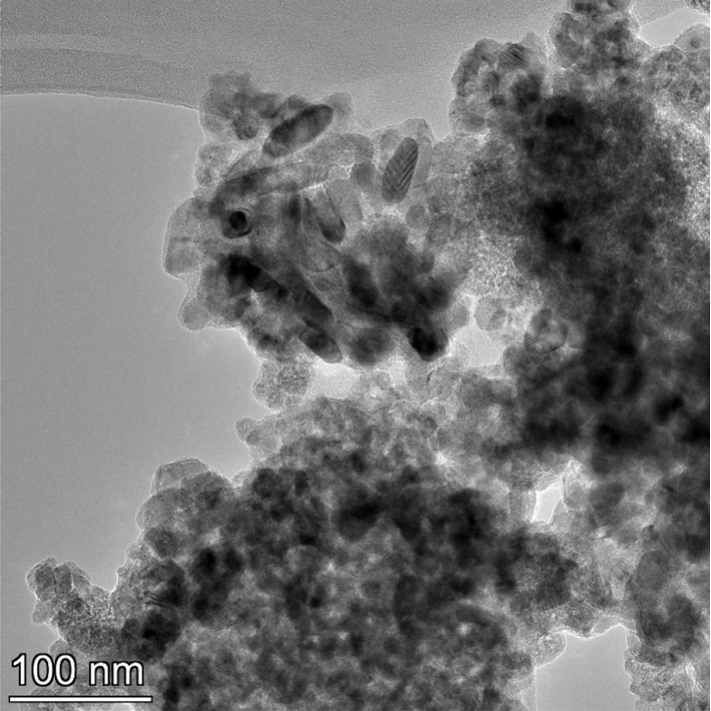
Powder population 5 with an average primary particle diameter of 23.538 nm ± 4.044 nm.

**Fig. 18:**
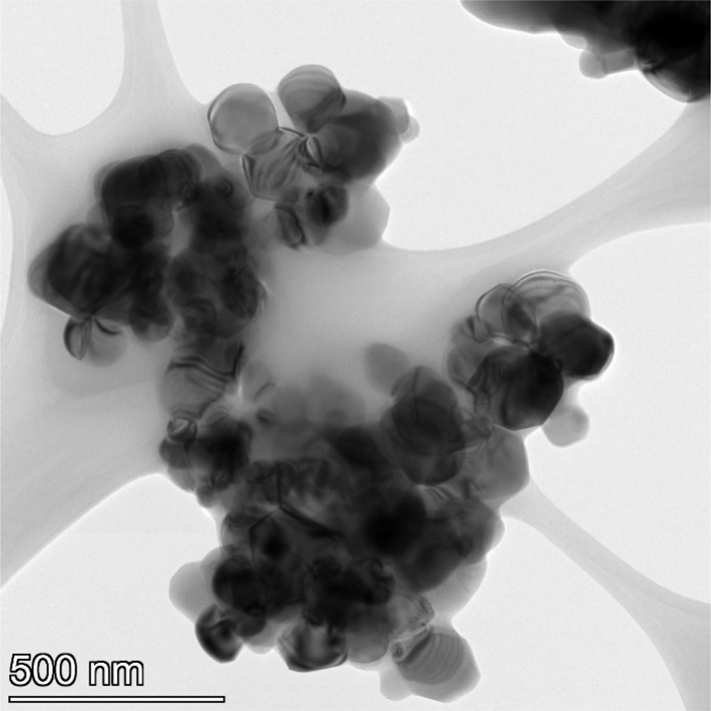
Powder population 6 with an average primary particle diameter of 126.002 nm ± 29.775 nm.

**Fig. 19:**
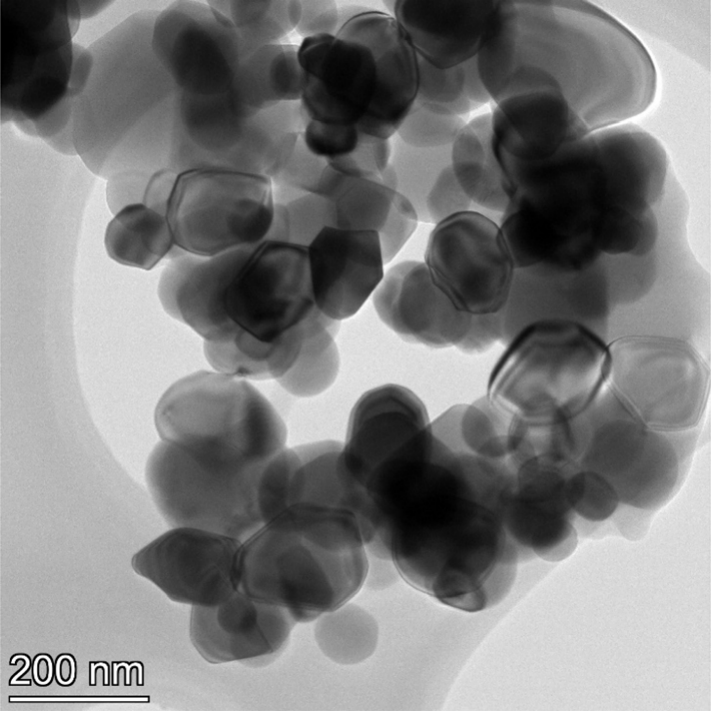
Powder population 7 with an average primary particle diameter of 142.614 nm ± 37.092 nm.

**Fig. 20:**
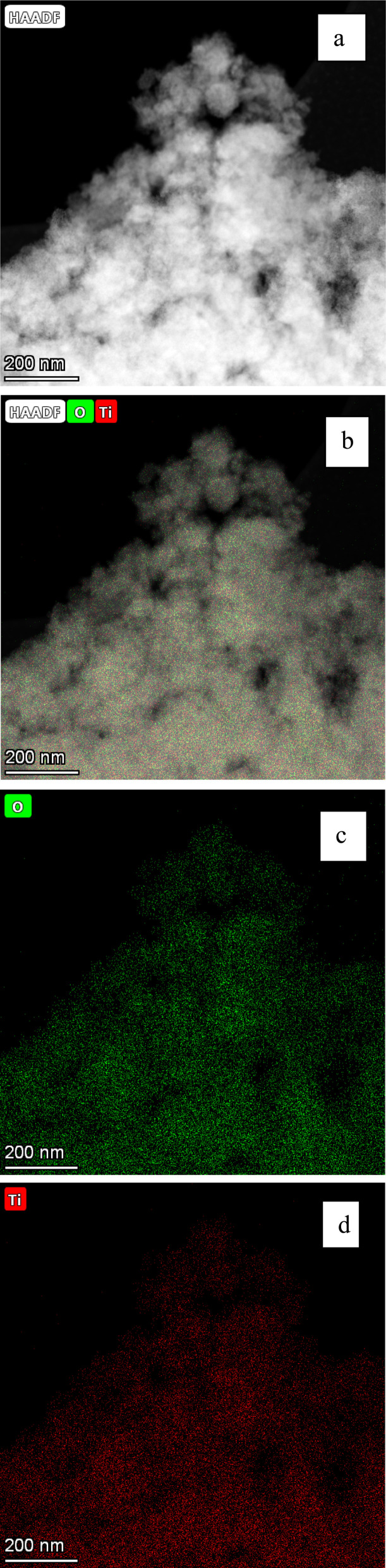
(a–d) Are the EDS micrographs of the powder population in Fig. 13. (b) The EDS micrographs for Ti^4+^ and O^2−^. (c) The EDS micrograph for O^2−^ and (d) the EDS micrograph for Ti^4+^ (d). No trace elements were found in figures (b)–(d).

#### B.2 Measured average primary particle diameters (Tables 3 and 4)

**Table 3:** The average primary particle diameter (nm) of each powder population using TEM from this work, and the BET (specific surface area) and aerodynamic article sizer average primary particle diameters from U.S. Nanomaterial Research and Spring Sky Nanomaterials

Powder population	Sample designation	Specific surface area (m^2^ g^−1^)	Spherical diameter from SSA (nm)	Aerodynamic particle sizer diameter (nm)	TEM avg. particle diameter^c^ (nm)	pH_PZC_
1	US3838^a^	480–650	3.22–2.38	5.00	5.307 ± 0527	1.73
2	US3490	200–240	7.73–6.44	18	12.689 ± 2.397	2.47
3	US3492	60	25.8	15	16.131 ± 3.958	3.04
4	US3493	40	38.7	35	21.108 ± 4.059	4.25
5	7910DL^b^	50–150	30.93–10.31	10–30	23.538 ± 4.044	5.46
6	US3411	85	18.2	100	126.002 ± 29.775	7.15
7	US1152M	10	154.6	800	142.02 ± 36.321	7.19

a U. S. nanomaterial research.

b Spring Sky nanomaterials.

c This work.

**Table 4:** Percentage difference values between the TEM measured average primary particle diameter and the vendor provided values^111,112^

Powder population	Percent variation using SSA (m^2^ g^−1^)	Percent variation aerodynamic particle sizer
1	39.325–55.154	5.785
2	39.081–49.247	41.855
3	59.941	7.011
4	22.229	28.936
5	31.930–56.199	57.516–27.454
6	149.515	20.632
7	8.858	463.301
Average value	51.148	81.561

Percentage difference between the TEM measured average primary diameters and the values using the Aerodynamic Particle Sizer and the values provided by each vendor.^112,113^Eqn (16) was then used to calculate the percentage difference between the TEM values and the BET (m^2^ g^−1^) and the values measured using the Aerodynamic Particle Sizer. The results are presented in Table 4.16
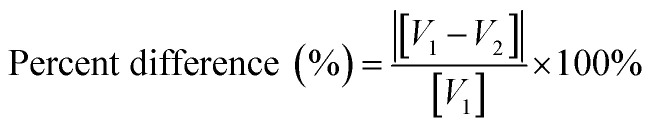
where *V*_1_ = TEM diameter (nm), *V*_2_ = BET (m^2^ g^−1^) or aerodynamic particle sizer diameter (nm).

### C Appendix C (Fig. 21)

**Fig. 21:**
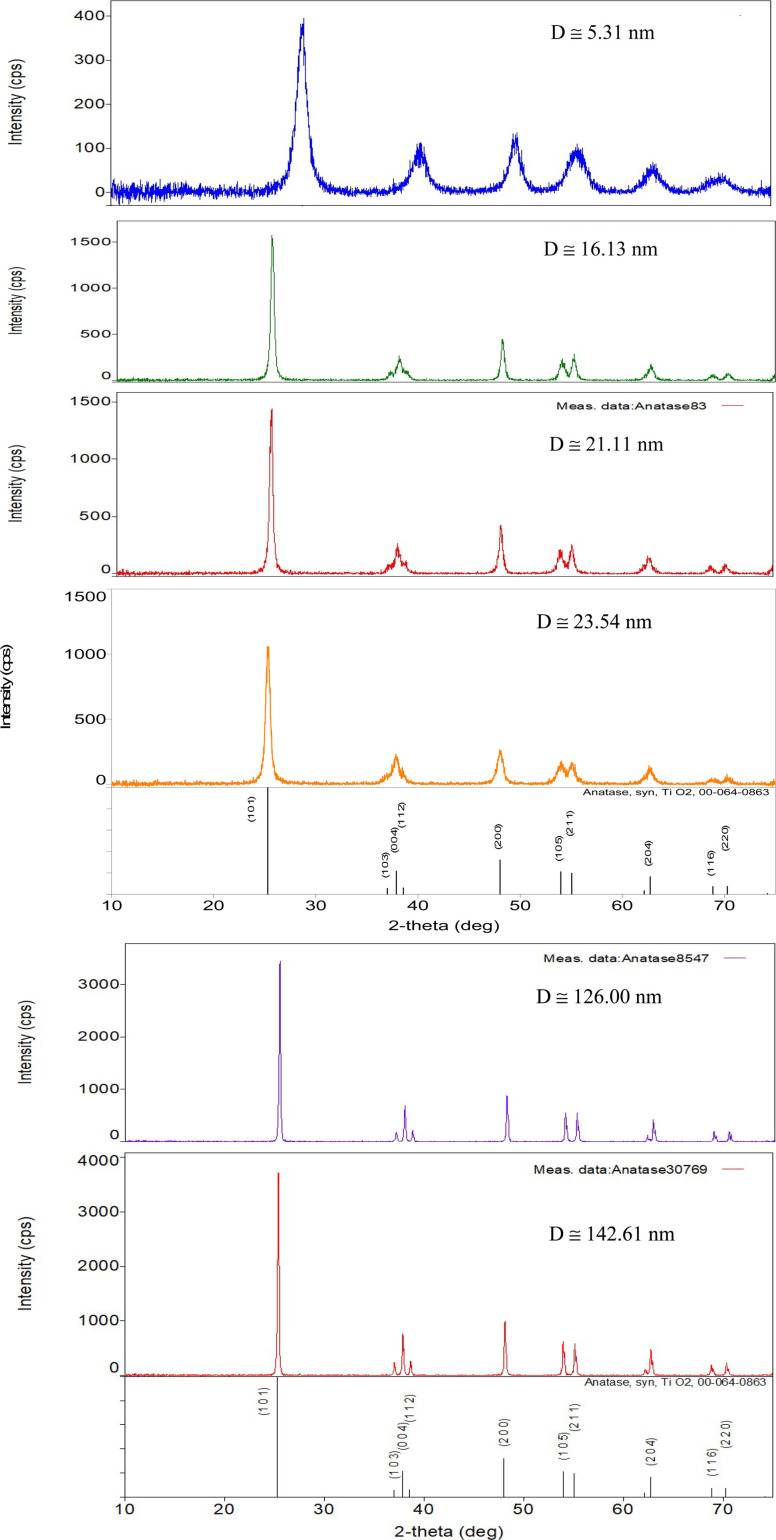
PXRD patterns for each of the samples used in this work, save Fig. 2. Each pattern fits the PXRD pattern JCPDS #00-064-0863 for synthesized anatase titania.^120^

### D Appendix D

#### D.1 Mass titration method (Fig. 22–27)

**Fig. 22:**
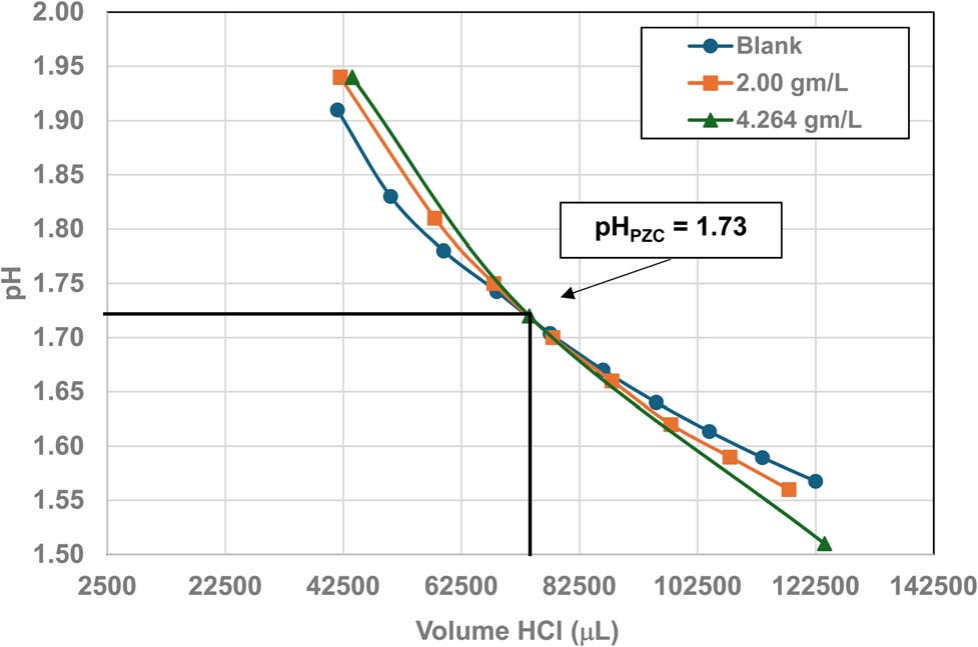
Mass titration curves of anatase titania with an average primary particle with a *d* ≅ 5.307 nm. Intersection of the three curves indicate the materials pH_PZC_ = 1.73. The large volume of HCl (0.01 N) titrant used was due to its low molar concentration.

**Fig. 23:**
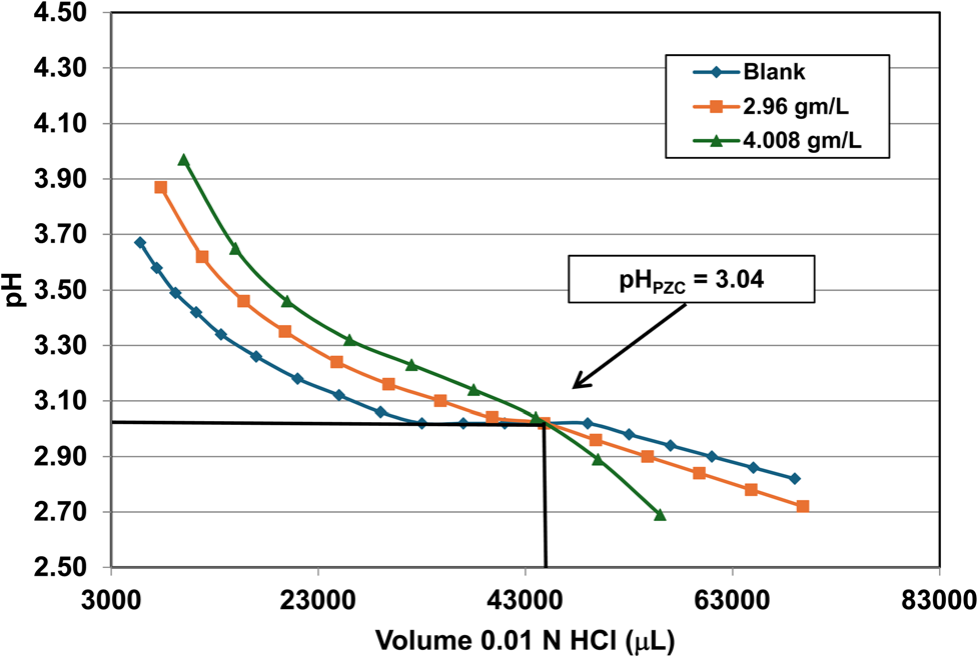
Mass titration curves of anatase titania with an average primary particle with a *d* ≅ 16.131 nm. Intersection of the three curves indicate the materials pH_PZC_ = 3.04. The large volume of HCl (0.01 N) titrant used was due to its low molar concentration.

**Fig. 24:**
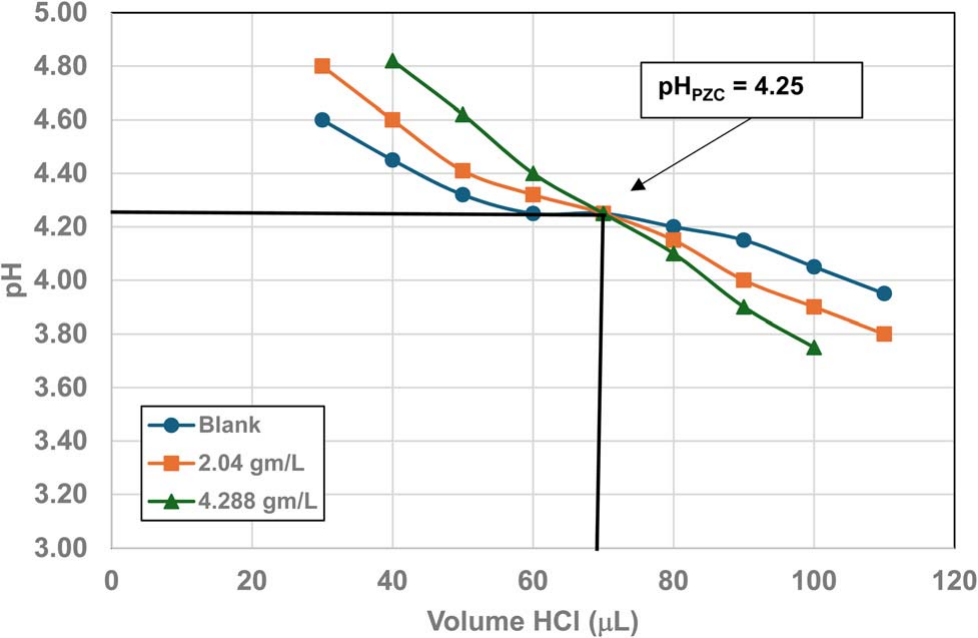
Mass titration curves of anatase titania with an average primary particle with a *d* ≅ 21.108 nm. Intersection of the three curves indicate the materials pH_PZC_ = 4.25.

**Fig. 25:**
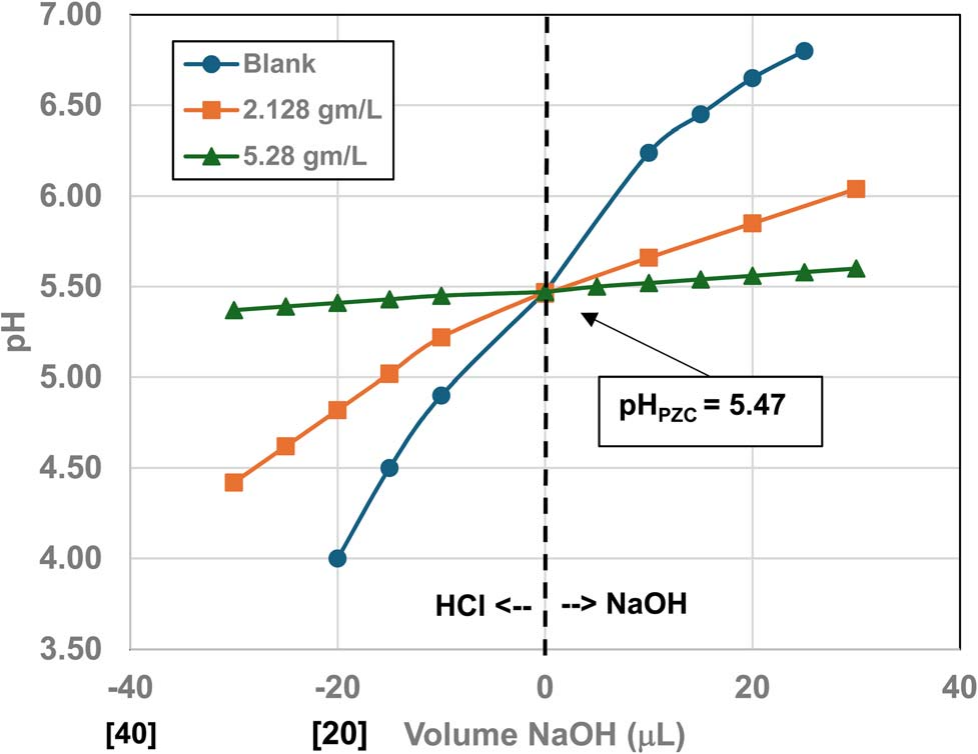
Mass titration curves of anatase titania with an average primary particle of *d* ≅ 23.538 nm. Intersection of the three curves indicates the materials pH_PZC_ = 5.46.

**Fig. 26:**
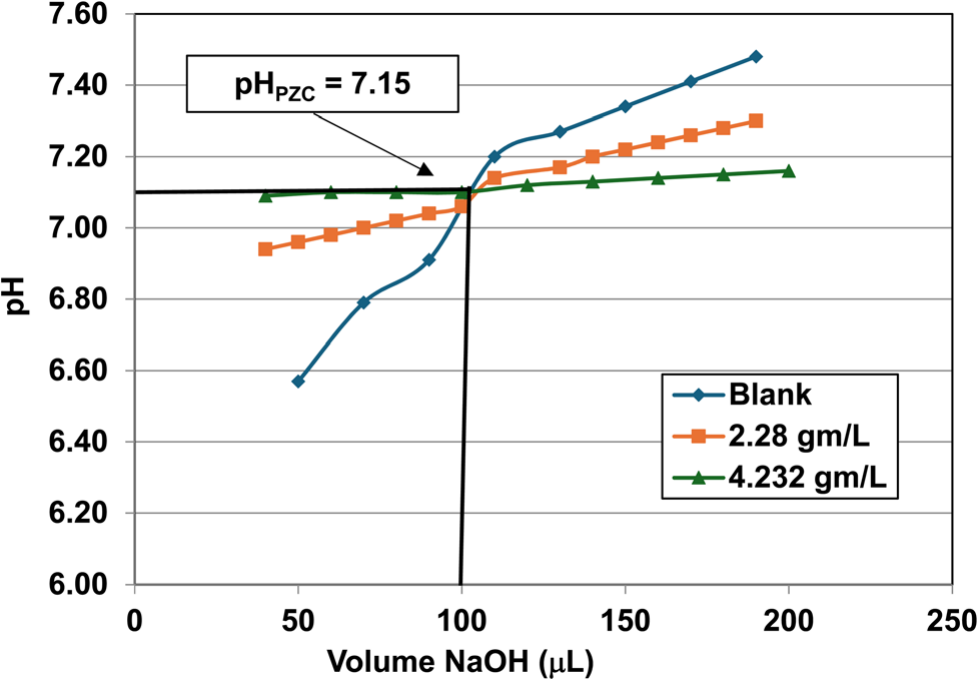
Mass titration curves of anatase titania with an average primary particle with a *d* ≅ 126.002 nm. Intersection of the three curves indicate the materials pH_PZC_ = 7.15.

**Fig. 27:**
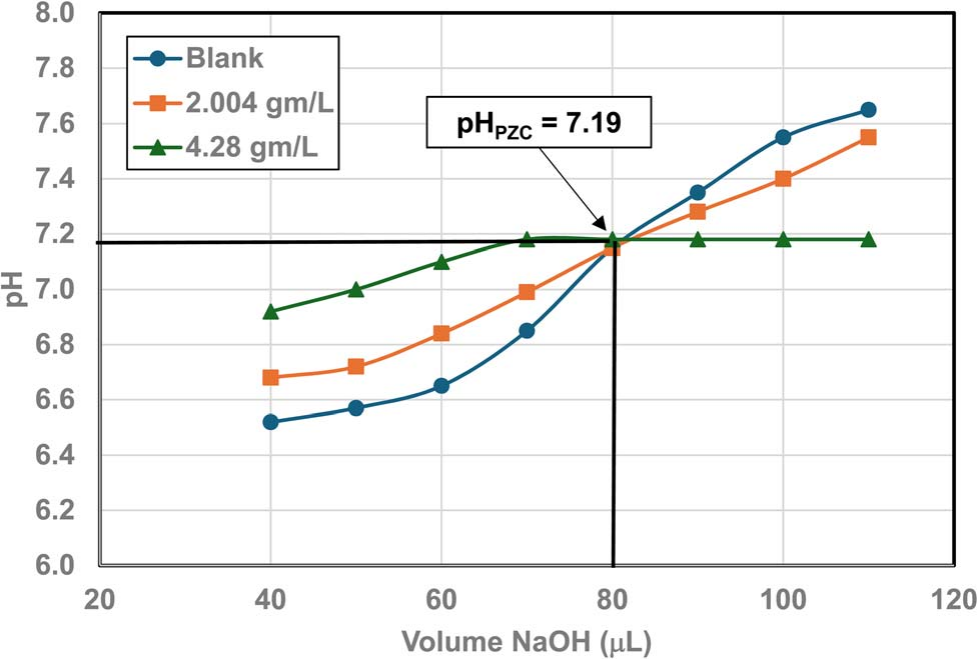
Mass titration curves of anatase titania with an average primary particle with a *d* ≅ 142.614 nm. Intersection of the three curves indicate the materials pH_PZC_ = 7.19.

Prior to each set of titration runs the HI 2214 pH/ORP meter, with an error ± 0.2 pH units (HI Manual) was calibrated using standard buffer solutions (BioPharm) at pH values of 4.0, 7.0 and 10.0. These are the pH set points required by the internal calibration program for this meter. The solution was stirred at low speed using a magnetic stir bar and stir plate (HI 190M): (1) which maintained a homogeneous sample concentration in the electrolyte solution and (2) properly dispersed the acid or base used to titrate the solution. The pH meter was fitted with both a pH and temperature probe, so that it could be used to measure both pH values and solution temperature concurrently during each run. Temperature, and pH, were monitored continuously and recorded concurrently after a given titrant volume was added and an equilibrium pH reached.

A volume of 250 mL 0.01 M KCl electrolyte solution was used in each run. It was prepared from a 1.0 M potassium chloride (KCl) solution (Aldon Corporation) and Reagent grade de-ionized water (DI) (ChemLab). A 600 mL Nalgene beaker was employed to avoid silica dissolving into the solution from a silica glass beaker^162^ and allow for large volumes of titrants. The anatase titania sample was taken directly from the manufacturer's container, measured on weighing paper using a GEM20 jeweler's scale (Smart Weigh), accurate to three significant figures, and then placed into the electrolyte solution. This procedure was used to minimize contamination due to handling.

The three runs consisting of samples sizes, 0.0 g L^−1^ (blank), ∼2.0 g L^−1^ and ∼4.0 g L^−1^ in the electrolyte. The pH was adjusted by hand using both a 200 µL and a 1000 µL Gilson pipettor. Each run was performed by only decreasing or increasing the pH of the system during each run. The titrants used were 0.01 N hydrochloric acid (HCl) (LabChem) or 0.2 N sodium hydroxide (NaOH) (LD Carlson Corporation). Interception of these three titration curves determined the materials PZC. The titrations were performed at ambient temperature and pressure. The solution acid molality/molarity was kept low to avoid problems with dissolution of the sample.^115^ This eliminated a situation where the surface dissolved and then reprecipitated back onto the surface of the particles during the titration runs.

Fig. 22 through 27 present the titration results of powder populations with average primary particle diameters of 5.307 nm, 16.131 nm, 21.108 nm, 23.538 nm, 126.002 nm, and 142.614 nm. The titration curve for the powder population with an average primary particle diameter of 12.689 nm is presented in Fig. 3. Titrations in Fig. 22 and 23 required between 6 to 7 hours. The shift in the direction in the titration curves in Fig. 25 and 26 from Fig. 22–24 was due to the direction of the titrant used.^163^ In Fig. 22–24 0.01 N HCl, was the titrant, whereas in Fig. 25–27 0.2 N NaOH was the titrant.

It was possible to achieve pH values below 2.0 pH units as the anatase titania is a photoelectric catalyst which splits water into O–H^−^ and H^+^.^164–166^ Therefore, based on the work by Brown *et al.*'s^167^ model of the Stern layer, Outer Helmholtz Plane (OHP) and diffuse region (*i.e.* solution) the O–H^−^ would be adsorbed onto the surface just outside the OHP to neutralize the Ti^4+^ surface atoms. At the same time the H^+^ ions would remain in the diffuse region (*i.e.* solution), resulting in a pH concentration below 2.0 pH units.

Fig. 25 is a combination of three sets of titrations (*i.e.* 6 individual titration curves). The initial pH values recorded for all 4 runs with a sample, before the titration began, were between the values of pH = 5.46–5.48. The first leg of the blank run was run first using 0.01 N HCl, and the second leg using 0.2 N NaOH. The first titration runs with a sample used 0.2 N NaOH. The second set of titrations with each sample were performed using 0.01 N HCl as the titrant. The weight for each run in the second set of titrations using 0.2 N NaOH was 0.0 g L^−1^, 2.129 g L^−1^, and 5.285 g L^−1^.

### E Appendix E

#### E.1 Results and interpretation of band gap measurements (Table 5, Fig. 28)

**Table 5:** The results of both sample preparations using reflection UV-VIS-Nir^119^

Sample diameter (nm)	Hydraulic press preparation (sample pressed at 10 tons)		XRD preparation (sample pressed at low pressure)	
	Cut off point (nm)	Bandgap (eV)	Cut off point (nm)	Bandgap (eV)
5.307	382.59	3.249	422.18	2.780
12.689	393.54	3.159	428.92	2.898
16.131	385.38	3.226	418.05	2.974
21.108	389.18	3.194	415.29	2.993
23.538	401.60	3.095	447.09	2.945
126.002	393.07	3.163	377.50	3.293
142.614	390.14	3.186	381.25	3.261

**Fig. 28:**
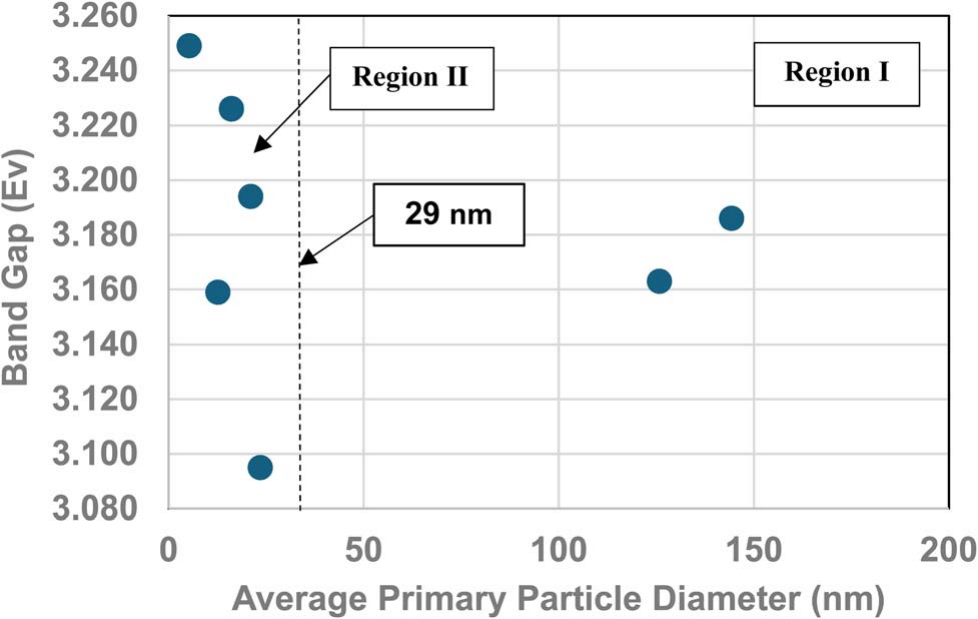
Indirect band gap values of anatase titania prepared by pressing each sample at 10 tons.^119^

Two methods were used to prepare the samples. The first used a mechanical press to apply 10 tons of pressure on the sample in a pellet mold. The second sample was hand pressed into a mold used to prepare X-ray diffraction samples at ambient temperature and pressure. The results of both these methods are presented in Table 5 for the reflection UV-VIS-NIR measurements.

A plot of the values in Fig. 28 demonstrates that the band gap values for Region II (5.307–23.538 nm) pressed at 10 ton were significantly different than those pressed at ambient pressure. For the samples pressed at 10 tons, band gap values decreased as the particle size increased in Region II (Fig. 28). This indicates that as the particle size decreased, surface bond lengths also decreased. For the hand pressed samples at ambient pressure resulted in the exact opposite trend. As particle size decreased in Region II, surface bond lengths increased [Fig. 5b]. Therefore, these results demonstrate the importance of maintaining the same physical conditions under which different techniques are used to examine a system. This allows for the correlation of different properties, when measured under identical experimental conditions.

## References

[cit1] Li T., Ciampi S., Darwish N. (2022). The Surface Potential of Zero Charge Controls the Kinetics of Diazonium Salts Electropolymerization. ChemElectroChem.

[cit2] Borghi F., Vyas V., Podesta A., Milani P. (2013). Nanoscale Roughness and Morphology Affect the Isoelectric Point of Titania. PLoS One.

[cit3] Akbarzadeh O., Zabidi N. A. M., Wahab Y. A., Hamizi N. A., Chowdhury Z. Z., Merican Z. M. A., Rahman M. A., Akhter S., Shalauddin M., Johan M. R. (2019). Effects of Cobalt Loading, Particle Size, and Calcination Condition on Co/CNT Catalyst Performance in Fischer Tropsch Reactions. Symmetry.

[cit4] Wang Y., Tejodor-Tejodor M. I., Tan W., Anderson M. A. (2016). Influence of Solution Chemistry on the Dielectric Properties of TiO_2_ Thin-Film Porous Electrodes. J. Phys. Chem. C.

[cit5] Tan W., Gao T., Wang Y. (2020). Influence of Surface Potential on the Capacitive Performance of the TiO_2_ Thin-Film Electrode with Different Crystalline Forms. Langmuir.

[cit6] Wang Y., Tejodor-Tejodor M. I., Tan W., Anderson M. A. (2016). Importance of Protons and Specifically Adsorbing Ions on Changing Capacitance, Space Charge Potential Inside the Solid, and the Interfacial Potential at TiO_2_ Aqueous Solution Interface. Electrochim. Acta.

[cit7] Yoon R. H., Salman T., Donnay G. (1979). Predicting Points of Zero Charge of Oxides and Hydroxides. Colloid Interface Sci..

[cit8] Bowker M. (2006). The surface structure of titania and the effect of reduction. Curr. Opin. Solid State Mater. Sci..

[cit9] Xia Y., Zhu K., Kaspar T. C., Du Y., Birmingham B. K., Park K. T., Zhang Z. (2013). Atomic Structure of the Anatase TiO_2_ (001) Surface. J. Phys. Chem. Lett..

[cit10] Parks G. A. (1965). The Isoelectric Points of Solid Oxides, Solid Hydroxides, and Aqueous Hydroxo Complex Systems. Chem. Rev..

[cit11] Linden D. and Reddy T. B., Handbook of Batteries, McGraw-Hill Companies Inc., Two Penn Plaza, New York, N. Y, 2002

[cit12] Carlson A. B. , Gisser D. G. and Manasse F. K., Electrical Engineering Concepts and Applications, Addison-Wesley Publishing Company, Inc., United States of America. 1981

[cit13] Rao S. R. and Leja J., Surface Chemistry of Froth Flotation, Volume 1: Fundamentals, Springer Science + Business Media, LLC, New York, 2nd edn, 2004

[cit14] Kaya A., Yukselen Y. (2005). Zeta-Potential of soils with surfactants and its relevance to electrokinetic remediation. J. Hazard. Mater. B.

[cit15] Khan S. K., Kar S. (2018). Surface charge is a function of organic carbon content and mineralogical compositions of soil. Eurasian J. Soil Sci..

[cit16] Bockris O. M. , Reddy A. K. N. and Gamboa-Aldeco M., Modern Electrochemistry, Volume 2, Fundamentals of Electrodics, Kluwer Academic, Plenum Publishers, New York, Boston, Dordrecht, London, Moscow, 2nd edn, 2000

[cit17] Sabbagh F., Muhamed I. I., Nazari Z., Peyman M., Katir N. M. (2018). Investigation of acyclovir-loaded, acrylamide-based hydrogels for potential use as vaginal ring. Mater. Today Commun..

[cit18] Hiemenz P. C. , Principles of Colloid and Surface Chemistry, Marcel Dekker, Inc., New York, Basel, 2nd edn, 1986

[cit19] Seiβ V., Thiel S., Eichelbaum M. (2022). Preparation and Real-World Applications of Titania Composite Materials for Photocatalytic Surface, Air, and Water Purification: State of the Art. Inorganics.

[cit20] Gomez R., Lopez T., Ortiz-Islas E., Navarrete J., Sanchez E., Tzompanztzi F., Bokhimi X. (2003). Effect of sulfation of the photoactivity of TiO_2_ sol-gel derived catalysis. J. Mol. Catal. A: Chem..

[cit21] Yogeswaran U., Chen S. M. (2008). A Review on the Electrochemical Sensors and Biosensors Composed Nanowires as Sensing Material. Sensors.

[cit22] Gupta K. K., Jassal M., Agrawal A. K. (2008). Sol-gel derived titanium dioxide finish of cotton fabric for self-cleaning. Indian J. Fibre Text. Res..

[cit23] Muhammad N., Zanon K. P. S., Iha M. N. Y., Ahmed S. (2018). The Use of Rutile- and Anatase-Titania Layers towards Light Scattering in Dye-Sensitized Solar Cells. ChemistrySelect.

[cit24] Azeez F., Al-Hetiani E., Arafa M., Abdelmonem Y., Nazeer A. A., Amin M. O., Madkour M. (2018). The effect of surface charge on photocatalytic degradation of methylene blue dye using chargeable titania nanoparticles. Sci. Rep..

[cit25] Zeng M. (2013). Influence of TiO_2_ Surface Properties on Water Pollution Treatment and Photocatalytic Activity. Bull. Korean Chem. Soc..

[cit26] Gul A., Ullah R., Sun J., Munir T., Bai S. (2021). The fabrication of TiO_2_–supported clinoptilolite *via* F^−^ contained hydrothermal etching and resultant highly energetic {0 0 1} facet for the enhancement of its photocatalytic activity. RSC Adv..

[cit27] Azeez F., Al-Hetlani E., Arafa M., Abdelmonem Y., Abdel Nazeer A., Amin O. M., Madkour M. (2018). The effect of surface charge on photocatalytic degradation of methylene blue dye using chargeable titania nanoparticles. Sci. Rep..

[cit28] Barisik M., Atalay S., Beskok A., Qian S. (2014). Size Dependent Surface Charge, Properties of Silica Nanoparticles. J. Phys. Chem. C.

[cit29] Palmer D. A., Machesky M. L., Benezeth P., Wesolowski D. J., Anovitz L. M., Deshon J. C. (2009). Adsorption of Ions on Zirconium Oxide Surfaces from Aqueous Solutions at High Temperatures. J. Solution Chem..

[cit30] Ananthapadmanabhan K. P., Somasundaran P. (1985). Surface Precipitation of Inorganic Surfactants and Its Role in Adsorption and Flotation. Colloids Surf..

[cit31] Gy P. , Sampling of Particulate Materials, Theory and Practice, Elsevier, New York, Revised 2^nd^ edn, 1982

[cit32] Gulicovski J. J., Cerovic L. S., Milonjic S. K. (2008). Point of Zero Charge and Isoelectric Point of Alumina. Mater. Manuf. Processes.

[cit33] Knapp C., Gil-Llambias J., Gulppi-Cabra M., Avila P., Blanco J. (1997). Phase Distribution in Titania–Sepiolite Catalyst Supports Prepared by Different Methods. J. Mater. Chem..

[cit34] Torres Sanchez R. M., Aglietti B. F., Porto Lopez J. M. (1988). PZC Modification of Mechanochemically Treated Kaolinite. Mater. Chem. Phys..

[cit35] Brookins D. G. , Eh-Ph Diagrams for Geochemistry, Springer-Vorlag, Berlin, Heidelberg, New York, London, Paris, Tokyo, 2012

[cit36] Preocanin T., Kallay N. (2006). Point of Zero Charge and Surface Charge Density of TiO_2_ in Aqueous Electrolyte Solution as Obtained by Potentiometric Mass Titration. Croat. Chem. Acta.

[cit37] Kosmulski M. , Chemical Properties of Material Surfaces, in Surfactant Science Series, ed. Hubbard, A. T., Marcel Dekker, Inc., New York, NY, vol. 102, 2001

[cit38] Kosmulski M. (2009). Compilation of PZC and IEP of sparingly soluble metal oxides and hydroxides from literature. Adv. Colloid Interface Sci..

[cit39] Suttiponparnit K., Jiang J., Sahu M., Suvachittanonot S., Charinpanitkul T., Biswas P. (2011). Role of Surface Area, Primary Particle Size, and Crystal Phase on Titanium Dioxide Nanoparticle Dispersion Properties. Nanoscale Res. Lett..

[cit40] Lin X., Li J., Ma S., Liu G., yang K., Tong M., Lin D. (2014). Toxicity of TiO_2_ Nanoparticles to *Escherichia Coli*: Effect of Particle Size, Crystal Phase and Water Chemistry. PLoS One.

[cit41] Hogg R. , Particle Technology Laboratory Lecture Notes and Laboratory Manual: Parts 1 and 2, Department of Mineral Engineering, Mineral Processing Section, The Pennsylvania State University, 1991

[cit42] Weast R. C. , Lide D. R., Astle M. J. and Beyer W. H., CRC Handbook of Chemistry and Physics, CRC Press, Inc., Boca Raton, FL, 70^th^ edn, 1989

[cit43] Leffler M. , Chemistry Dept., University of Connecticut, Unpublished results, 2021

[cit44] Kosmulski M., Durand-Vidal S., Maczka E., Rosenholm J. B. (2004). Morphology of synthetic goethite particles. J. Colloid Interface Sci..

[cit45] Piazza R. D., Pelizaro T. A. G., Rodriguez-Chanfrau J. E., La Serna A. A., Veranes-Pantoja Y., Guastaldi A. C. (2020). Calcium phosphates nanoparticles: The effect of freeze-drying on particle size reduction. Mater. Chem. Phys..

[cit46] Cavajda V., Uhlik P., Derkowski A., Caplovicova M., Madejova J., Mikula M., Ifka T. (2015). Influence of grinding and sonication on the crystal structure of talc. Clays Clay Miner..

[cit47] Botella R., Chiter F., Costa D., Nakashima S., Lefevre G. (2021). Influence of hydration/dehydration on adsorbed molecules: Case of phthalate on goethite. Colloids Surf., A.

[cit48] Su C., Suarez D. L. (2000). Selenate and Selenite Sorption on Iron Oxides: An Infrared and Electrophoretic Study. Soil Sci. Soc. Am. J..

[cit49] Borer P. M., Sulzberger B., Reichard P., Kraemer S. M. (2005). Effect of siderophores on the light-induced dissolution of colloidal iron (III) (hydr)oxides. Mar. Chem..

[cit50] Rennert D. G. T. , Sorption of Iron-Cyanide Complexes on Iron Oxides and in Soils, Ph. D. thesis, Ruhr-Universität Bochum, Germany, 2002

[cit51] Kersten M., Vlasova N. (2009). Arsenite Adsorption on goethite at elevated temperatures. Appl. Geochem..

[cit52] Dabiza A., Kersten M. (2020). Exothermic Adsorption of chromate by goethite. Appl. Geochem..

[cit53] Herrera Ramos A. C., McBride M. B. (1996). Goethite Dispensability in Solutions of Variable Ionic Strength and Soluble Organic matter Content. Clays Clay Miner..

[cit54] Regenspurg S. , Characterization of Schwermannite–Geochemical Interactions with Arsenate and Chromate and Significance in Sediments of Lignite Opencast Lakes, Ph. D. thesis, University of Bayreuth, Germany, 2002

[cit55] Zhang J. S., Stanforth R., Pehkonen S. O. (2007). Proton-arsenic adsorption ratios and zeta potential measurements: Implications for protonation of hydroxyls on the goethite surface. J. Colloid Interface Sci..

[cit56] Gaboriaud G., Ehrhardt J. J. (2003). Effects of different crystal faces on the surface charge of colloidal goethite (α−FeOOH) particles: An experimental and modeling study. Geoch. Cosm. Act..

[cit57] Kosmulski M. and Mączka E., Surface Charges at Goethite–Electrolyte Solution Interface, VII Ogólnopolska 10 Konferencja Naukow, 2013

[cit58] Liu J., Zhu R., Ma L., Fu H., Lin X., Parker S. C., Molinari M. (2021). Adsorption of phosphate and cadmium on iron (oxyhdr)oxides: A comparative study on ferrihydrite, goethite, and hematite. Geoderma.

[cit59] Luxton T. P., Tadanier C. J., Eick M. J. (2006). Mobilization of Arsenite by Competitive Interaction with Silicic Acid. Soil Sci. Soc. Am. J..

[cit60] Shenk A. , Calculus and Analytic Geometry, Goodyear Publishing, Inc., Santa Monica, California, 2nd edn, 1979

[cit61] Lee S., Paik U., Hacklye H. A., Jung Y. G., Yoon K. J. (2004). Microstructure and permittivity of sintered BaTiO_3_; influence of particle surface chemistry in an aqueous medium. Mater. Res. Bull..

[cit62] Shih W. H., Kisailus D., Wei Y. (1995). Silica coating on barium titanate particles. Mater. Lett..

[cit63] Yu B. Y., Wei W. C. J., Hsu K. C. (2004). Study of processing adsorption mechanism of amphoteric polyelectrolyte in BaTiO_3_ colloids suspension. J. Chem. Res..

[cit64] Lee B. I. (1998). Electrokinetic Behavior of Barium Titanate Powders in Water. J. Korean Phys. Soc..

[cit65] Chen Z. C., Ring T. A., Lamaitre J. (1992). Stabilization and Processing of Aqueous BaTiO_3_ Suspensions with Polyacrylic Acid. J. Am. Ceram. Soc..

[cit66] De Laat A. W. M., van den Heuvel G. L. T. (1993). Competitive and displacement adsorption of polyvinyl alcohol and the ammonium salt of a polyacrylic acid on BaTiO_3_. Colloids Surf., A.

[cit67] de Laat A. W. M., Derks W. P. T. (1993). Colloidal stabilization of BaTiO_3_ with poly (vinyl alcohol) in water. Colloids Surf., A.

[cit68] Tripathy S. S., Raichur A. M. (2011). Dissolution of BaTiO_3_ nanoparticles in aqueous suspensions. J. Exp. Nanosci..

[cit69] Paik U. (2000). Influence of Solids Concentration on the Isoelectric Point of Aqueous Barim Titanate. J. Am. Ceram. Soc..

[cit70] Bergna H. E. and Burn I., Ceramic Dielectric Compositions of and Method for Improving Sinterability, US Pat.5001804, 1991

[cit71] Gheradrdi P., Matijevic E. (1998). Homogeneous Precipitation of Spherical Colloidal Barium Titanate Particles. Colloids Surf..

[cit72] Paik U., Hackley B. A., Choi S. C., Jung Y. G. (1998). The effect of electrostatic repulsive forces on the stability of BaTiO_3_ particles suspended in non-aqueous media. Colloids Surf., A.

[cit73] Leong Y. K. (1999). Interparticle forces arising from an adsorbed strong polyelectrolyte in colloidal dispersions: charged patch attraction. Colloid Polym. Sci..

[cit74] Dey S., Bhattacharya P., Bandyopadhyay S., Roy S. N., Majumdar S., Sahoo G. C. (2018). Single Step Preparation of Zirconia Ultrafiltration Membrane over Clay-Alumina Based Multichannel Ceramic Support for Waste Treatment. J. Membr. Sci. Res..

[cit75] Eibl M., Virtanen S., Pischel F., Bok F., Lonnrot S., Shaw S., Huittinen N. (2019). A spectroscopic study of trivalent cation (Cm^3+^ and Eu^3+^) sorption on monoclinic zirconia (ZrO_2_). Appl. Surf. Sci..

[cit76] Panalytical M. and Slips C., The Importance of Particle Size Analysis and Zeta Potential, AZoNano, https://www.azonano.com/article.aspx?ArticleID=1097, 2024

[cit77] Leong Y. K., Scales P. J., Healy T. W., Boger D. V. (1993). Rheological Evidence of Adsorbate-mediated Short-range Steric Forces in Concentrated Dispersions. J. Chem. Soc., Faraday Trans..

[cit78] Biggs S., Healy T. W. (1994). Electrosteric Stabilization of Colloidal Zirconia with Low-molecular-weight Polyacrylic Acid. J. Chem. Soc., Faraday Trans..

[cit79] Ryan J. N., Elimelech M., Baseseman J. L., Magelky R. D. (2000). Silica-Coated Titania and Zirconia Colloids for Subsurface Transport Field Experiments. Environ. Sci. Technol..

[cit80] Dering A. , Catalyst Preparation Uptake of Gold onto Different Supports, Project Report, Laboratory for Product and Process Design, University of Illinois at Chicago, 2004

[cit81] Gnann K. A. , Predicting Zeta Potential Ad Adsorption Behavior in the Al_2_O_3_-SiO_2_-TiO_2_-ZrO_2_-Surfactant System, Masters thesis, Alfred University, 2004

[cit82] Hristovski K. D., Westerhoff P. K., Crittenden J. C., Olson L. W. (2008). Environ. Sci. Technol..

[cit83] Marcos P. J. B., Castro R. H. R., Gouvea C. D. (2001). Study of zirconia and magnesia suspensions in ethanol. Ceramica.

[cit84] Wang J., Gao L., Sun J., Li A. (1999). Surface Characterization of NH_4_PAA-Stabilized Zirconia Suspensions. J. Colloid Interface Sci..

[cit85] Leffler M. , Chemistry Dept. University of Connecticut, Unpublished Results, 2004

[cit86] Ramos-Tejada M. M., Ontiveros A., Viota J. L., Duran J. D. G. (2003). Interfacial and rheological properties of humic acid/hematite suspensions. J. Colloid Interface Sci..

[cit87] Jeon B. H., Dempsey B. A., Burgos W. D., Royer R. A., Roden E. E. (2004). Modeling the sorption kinetics of divalent metal ions to hematite. Water Res..

[cit88] Reiller P., Moulin V., Casanova F., Dautel C. (2002). Retention behavior of humic substances onto mineral surfaces and consequences upon thorium (IV) mobility: case of iron oxides. Appl. Geochem..

[cit89] Plaza R. C., Duran J. D. G., Quirantes A., Ariza M. J., Delgado A. V. (1997). J. Colloid Interface Sci..

[cit90] Arai Y., Sparks D. L., Davis J. A. (2004). Effects of Dissolved Carbonate on Arsenate Adsorption and Surface Speciation at the Hematite-Water Interface. Environ. Sci. Technol..

[cit91] Lenhart J. J., Honeyman B. D. (1999). Reactions at the Solid/Solution Interface Fe-Oxides and Hydroxides. Geochim. Cosmochim. Acta.

[cit92] Lytle D. A. , Payne J. M. and Song T. J., Adsorption Media for Arsenic Removal, U. S. Environmental Protection Agency, ORD, NRMRL, WSWRD, TTEB, Cincinnati, Ohio 45268, 2004

[cit93] Tombacz E., Libor A., Illes E., Majzik A., Klumpp E. (2004). The role of reactive surface sites and complexation by humic acids in the interaction of clay mineral and iron oxide particles. Org. Geochem..

[cit94] Xu C. Y., Deng K. Y., Li J. Y., Xu R. K. (2015). Impact of environmental conditions on aggregation kinetics of hematite and goethite nanoparticles. J. Nanopart. Res..

[cit95] Pivovarov S. (2001). Adsorption of Cadmium onto Hematite: Temperature Dependence. J. Colloid Interface Sci..

[cit96] Fengqiu T., Xiaoxian H., Yufeng Z., Jingkun G. (2000). Effect of dispersants on surface chemical properties of nano-zirconia suspensions. Ceram. Int..

[cit97] Kim M. J., Yang T. Y., Lee Y. B., Park H. C. (2002). Dispersion stability of Y-TZP/Ce-TZP powder system and slip casting. J. Mater. Sci..

[cit98] Weng M. T., Wei W. C., Huang C. Y. (2007). Influence of 3Y-TZP on Microstructure and Mechanical Properties of Al_2_O_3_-based Composites. Key Eng. Mater..

[cit99] Bleier A. and Omatete O. O., Rheology and Microstructure of Concentrated Zirconia-Alumina Suspensions for Gelcasting Composites, Oak Ridge National Laboratory, P. O. Box 2008, Oak Ridge, TN. 37831, 1992

[cit100] Wei-Cheng J. W., Wang S. C., Ho F. Y. (1999). Electrokinetic Properties of Colloidal Zirconia Powders in Aqueous Suspension. J. Am. Ceram. Soc..

[cit101] Sanchez-Herencia A. J., Pascual C., He J., Lange F. F. (1999). ZrO_2_/ZrO_2_ Layered Composites for Crack Bifurcation. J. Am. Ceram. Soc..

[cit102] Tan Q., Tang Z., Zhang A., Yao W., Fang K. (2003). Optimization of the rheological properties of nanometer sized tetragonal polycrystal zirconia slurries for aqueous-gel-tape-casting processing. Mater. Sci. Eng. B.

[cit103] Chen C. C., Hsiang H. I., Hsu S. W. (2008). Preparation and Characterization of Y-TZP powders coated with alumina. J. Ceram. Process. Res..

[cit104] Wang J., Gao L., Sun J. (1999). Interface Adsorption of Y-TZP Aqueous Suspensions. J. Inorg. Mater..

[cit105] Ferrari B., Moreno R. (2000). Zirconia Thick Films Deposited on Nickel by Aqueous Electrophoretic Deposition. J. Electrochem. Soc..

[cit106] Kong Y. M., Kim S., Kim H. E. (1999). Reinforcement of Hydroxyapatite Bioceramic by Addition of ZrO_2_ Coated with Al_2_O_3_. J. Am. Ceram. Soc..

[cit107] Pagnoux C. (2002). Suspension systems for coagulation processing. J. Ceram. Process. Res..

[cit108] Shojai F., Pettersson A., Mantyla T. A., Rosenholm J. B. (2000). Detection of carbon residue on the surface of 3Y-ZrO_2_ powder and its effect on the rheology of the slip. Ceram. Int..

[cit109] Shojai F., Pettersson A. B. A., Mantyla T., Rosenholm J. B. (2000). Electrostatic and electrostatic stabilization of aqueous slips of 3Y–ZrO_2_ powder. J. Eur. Ceram. Soc..

[cit110] Shannon R. D. (1976). Revised effective ionic radii and systematic studies of inter-atomic distances in halides and chalcogenides. Acta Crystallogr. A.

[cit111] Lin H., Huang C. P., Li W., Ni C., Ismat Shah S., Tseng Y. H. (2006). Size dependency of nanocrystalline TiO_2_ on its optical property and photocatalytic reactivity exemplified by 2-chlorophenol. Appl. Catal., B.

[cit112] US Research Nanomaterials, Inc. us-nano.com, 3302 Twig Leaf Lane, Houston, TX 77084, USA. Phone: (Sales) +832-359-7887; Fax: +281-492-8628

[cit113] SkySpring Nanomaterials, Inc. 2935 Westhollow Drive, Houston, TX, 77082, USA Phone: +281-870-1700, Fax: +281-870-8002, Email: sales@ssnano.com, 2020

[cit114] Van Gestel T., Vandecasteele C., Buekenhoudt A., Dotremont C., Luyten J., Van der Brugger B. (2003). Corrosion properties of alumina and titania NF membranes. J. Membr. Sci..

[cit115] HMC Harmony Chemical, Resources, Titanium Dioxide Chemical Properties, https://www.ti-line.net/resources/titanium-dioxide-properties/titanium-dioxide-chemical-properties.html, 2024

[cit116] Scimeca M., Bischetti S., Lamisra H. K., Bonfiglio R., Bonanno E. (2018). Energy Dispersive X-Ray (EDX), microanalysis: A powerful tool in biomedical research and diagnosis. Eur. J. Histochem..

[cit117] https://www.rigaku.com/applications/bytes/xrd/ultima-iv/1864928622, 2024

[cit118] Mahmood T., Saddiue M. T., Naeem A., Westerhoff P., Mustafa S., Alum A. (2011). Comparison of Different Methods for the Point of Zero Charge Determination of NiO. Ind. Eng. Chem. Res..

[cit119] Le Clercq R. and Beelen D., Analysis Report: ReportY0LFP098UV_Rev00.Docx, Bandgap Measurements on Nanoscale Anatase Titanium Oxide Powders (Cc Ok 6/23) Eurofins Materials Science Netherlands B.V, 2020

[cit120] International Center for Diffraction Data, 12 Campus Blvd., Newtown Square, PZ 19073, USA. JCPDS file #00-0634-0863

[cit121] Hughes C. E., Manjunatha Reddy G. N., Masiero S., Brown S. P., Wiliams P. A., Harris K. D. M. (2017). Determination of a complex crystal structure in the absence of single crystals: analysis of powder X-ray diffraction data, guided by solid-state NMR and periodic DFT calculations, reveals a new 20-deoxyguanosine structural motif. Chem. Sci..

[cit122] Alexzevalkink A. , https://wordpress.com/wpcontent/uploads/2017/04/me133652.pdfSmartLab, Guidance Reference Model, Rigaku Corporation

[cit123] Samad J. E., Hashim S., Ma S., Regalbuto J. R. (2014). Determining surface composition of mixed oxides with pH. J. Colloid Interface Sci..

[cit124] Teh E. J., Leong Y. K., Craig V. S. J. (2017). Surface Forces and Rheology of Titanium Dioxide in the Presence of Dicarboxylic Acids: From Molecular Interactions to Yield Stress. Langmuir.

[cit125] Amano F., Yasumoto T., Mahaney O. O. P., Uchida S., Shibayama T., Terada Y., Ohtani B. (2010). Highly Active Titania Photocatalyst Particles of Controlled Crystal Phase, Size and Polyhedral Shapes. Top. Catal..

[cit126] Ko K. H., Lee Y. C., Jung Y. J. (2005). Enhanced efficiency of dye-sensitized TiO_2_ solar cells (DSSC) by doping of metal ions. J. Colloid Interface Sci..

[cit127] Samsudin E. M. B. , Improved Titanium Dioxide Promoted Photocatalyst for Degradation on Pollutant in Water, PhD thesis, Institute of Graduate Studies, University of Malaya, Kualalumpur, 2016

[cit128] Lee J. W., Othman M. R., Eom Y., Lee T. G., Kim W. S., Kim J. (2008). The effects of thermally treated SiO_2_/TiO_2_ sperhical core-shell particles for photo-catalysis of methyl orange. Microporous Mesoporous Mater..

[cit129] Jegadeesan G., Al-Abed S. R., Sundaram V., Choi H., Scheckel K. G., Dionysious D. D. (2010). Arsenic sorption of TiO_2_ nanoparticles: Size and crystallinity effects. Water Res..

[cit130] Mandzy N., Grulke E., Druffel T. (2005). Breakage of TiO_2_ agglomerates in electrostatically stabilized aqueous dispersion. Powder Technol..

[cit131] Zeng M. (2013). Influence of TiO_2_ Surface Properties on Water Pollution Treatment and Photocatalytic Activity. Bull. Korean Chem. Soc..

[cit132] Jukic J., Juracic T., Begovic T. (2024). Effects of polyion adsorption on surface properties of TiO_2_. Adsorption.

[cit133] Tarafdar A., Raliya R., Wang W. N., Biswas P., Tarafdar J. C. (2013). Green Synthesis of TiO_2_ Nanoparticle Using *Aspergillus tubingensis*. Adv. Sci., Eng. Med..

[cit134] Leffler M. , Chemistry Dept, University of Connecticut, Unpublished Results, 2004

[cit135] Lim C. S. (2010). Synthesis and characterization of TiO_2_-ZnO nanocomposite by a two-step chemical method. J. Ceram. Process. Res..

[cit136] Lin H., Huang C. P., Li W., Ni C., Ismat Shah S., Tseng Y. H. (2006). Size dependency of nanocrystalline TiO_2_ on its optical property and photocatalytic reactivity exemplified by 2-chlorophenol. Appl. Catal., B.

[cit137] O'Donnell K. P., Chen X. (1991). Temperature Dependence of Semiconductor Band Gap. Appl. Phys. Lett..

[cit138] Wei W., Hong-Lie S., Jia-Le J., Jin-Ze L., Yue M. (2015). Effect of thermal pretreatment of metal precursor on the properties of Cu_2_ZnSnS_4_ films. Chin. Phys. B.

[cit139] Dai S., Wu Y., Sakai T., Du Z., Sakai H., Abe M. (2010). Preparation of Highly Crystalline TiO_2_Nanostructures by Acid-assisted Hydrothermal Treatment of Hexagonal-structured Nanocrystalline Titania/Cetyltrimethyammonium Bromide Nanoskeleton. Nanoscale Res. Lett..

[cit140] Stengl V., Houskova V., Bakardijeva S., Murafa N. (2010). Photocatalytic Activity of Boron-Modified Titania under UV and Visible-Light Illumination. ACS Appl. Mater. Interfaces.

[cit141] Long H., Yang G., Chen A., Li Y., Lu P. (2009). Femtosecond Z-scan measurement of third-order optical nonlinearities in anatase TiO_2_ thin films. Opt. Commun..

[cit142] Kernazhitsky L., Shymanovska V., Gavrilko T., Naumov V., Kshnyakin V., Ukr J. (2012). Optical absorption of polydisperse TiO_2_: Effect of surface doping by transition metal cations. Phys. Opt..

[cit143] Reddy K. M., Manorama S. V., Reddy A. R. (2002). Bandgap Studies on Anatase Titanium DiOxide Nanoparticles. Mater. Chem. Phys..

[cit144] Serpone N., Lawless D., Khairutdinov R. J. (1995). Size Effects on the Photophysical Properties of Colloidal Anatase TiO_2_ Particles: Size Quantization of Direct Transitions in This Indirect Semiconductor. Phys. Chem..

[cit145] Bedikyan L., Zakhariev S., Zakhariev M. (2013). Titanium Dioxide Thin Films: Preparation and Optical Properties. J. Chem. Technol. Metall..

[cit146] Bal I., Baykul M. C., Sarac U. (2021). The Effect of Solution Temperature on Chemically Manufactured CdS Samples. Chalcogenide Lett..

[cit147] Szekeres M., Tombacz E. (2012). Surface charge characterization of metal oxides by potentiometric titration, revisited theory and experiment. Colloids Surf., A.

[cit148] Tuckerman M. , Vitz E., Moore J. W., Shorb J., Prat-Resina X., Wendorff T., Hahn A., Lancashire R. J. and Contributors, Electronegativity and Dipole Moment, Physical Chemistry For The Biosciences, 2020, https://chem.libretexts.org/@go/page/41382

[cit149] Lee H. J., Jamison A. C., Lee T. R. (2015). Surface Dipoles: A Growing Body of Evidence Supports Their impact and Importance. Acc. Chem. Res..

[cit150] Yan W., Li S., Zhang Y., Yao Q. (2010). Effects of Dipole Moments and Temperature on the Interaction Dynamics of Titania Nanoparticles during Agglomeration. J. Phys. Chem. C.

[cit151] Sun C. Q., Li L., Tay B. K., Huang H. (2001). An extended ‘quantum confinement’ theory: Surface-coordination imperfection modifies the entire band structure of a nanosolid. J. Phys. D: Appl. Phys..

[cit152] Pauling L. , The Nature of the Chemical Bond, Cornell University Press, Ithaca New York, 3^rd^ edn, 1960

[cit153] Bosco B. R. J. and Jeyaprakash B. G., Melting points, mechanical properties of nanoparticles and Hall Petch relationship for nanostructured materials, NPTEL, pp. 1–18

[cit154] Font F., Myers T. G. (2013). Spherically symmetric nanoparticle melting with a variable phase change temperature. J. Nanopart. Res..

[cit155] Hu W. , Xiao S., Deng H., Luo W. and Deng L., Thermodynamic properties of nano-silver and alloy particles, Department of Applied Physics, Hunan University, Changsha 410082, PR China, 2010, pp. 1–34

[cit156] Jones M. , Ignition and Combustion Characteristics of Nanoscale Metal and Metal Oxide Additives in Biofuel (Ethanol) and Hydrocarbons, Ms thesis, The University of Toledo, 2011 10.1186/1556-276X-6-246 PMC3211307 21711760

[cit157] Qu Y. D., Liang X. L., Kong X. Q., Zhang W. J. (2017). Size-dependent cohesive Energy, Melting Temperature, and Debye Temperature of Spherical metallic Nanoparticles. Phys. Met. Metallogr..

[cit158] Gibbs G. V., Hill F. C., Boisen M. B., Downs R. T. (1998). Power law relationships between bond length, bond strength and electron density distributions. Phys. Chem. Miner..

[cit159] Livey D. T., Murray P. (1956). Surface Energies of Solid Oxides and Carbides. J. Am. Ceram. Soc..

[cit160] Oliver P. M., Watson G. W., Kelsey E. T., Parker S. C. (1997). Atomistic simulation of the surface structure of the TiO_2_ polymorphs rutile and anatase. J. Mater. Chem..

[cit161] Sears F. W. , Zemansky M. W. and Young H. D., University Physics, Fifth Edition, Addison-Wesley Publishing Company, Inc. Reading, Massachusetts, Menlo Park, California, London, Amsterdam, Don Mills, Ontario, Sydney, 1980

[cit162] Smith B. , NOAH, 212 Rogers Ave, Milford, CT 06460, Personal Communication, 2016

[cit163] Sunjuk M., Arar H., Mahmoud W. F., Majdalawi M., Krishan M. M., Salha Y. A., El-Eswed B. (2019). Adsorption of cationic and anionic organic dyes of SiO_2_/CuO composite. Desalin. Water Treat..

[cit164] Zhao W. N., Liu Z. P. (2014). Mechanism and active site of photocatalytic water splitting on titania in aqueous surroundings. Chem. Sci..

[cit165] Wei B., Calatayud M. (2022). Hydrogen activation on Anatase TiO_2_: Effect of surface termination. Catal. Today.

[cit166] Eidsvag H., Bentouba S., Vajeeston P., Yohi S., Velauthapillai V. (2021). TiO_2_ as a Photocatalyst for Water Splitting—An Experimental and Theoretical Review. Molecules.

[cit167] Brown M. A., Abbas Z., Kleibert A., Green R. G., Goel A., May S., Squires T. M. (2016). Determination of Surface Potential and Electrical Double-Layer Structure at the Aqueous Electrolyte-Nanoparticle Interface. Phys. Rev. X.

